# Forest connectivity boosts carbon recovery in regenerating Atlantic Forests

**DOI:** 10.1038/s43247-026-03480-5

**Published:** 2026-04-09

**Authors:** Thais M. Rosan, Laura B. Vedovato, Viola H. A. Heinrich, Celso H. L. Silva-Junior, Pedro H. S. Brancalion, Stephen Sitch, Luiz E. O. C. Aragão

**Affiliations:** 1 Faculty of Environment, Science and Economy, University of Exeter, Exeter, UK; 2 Department of Forest Sciences, “Luiz de Queiroz” College of Agriculture, University of São Paulo, Piracicaba, Brazil; 3 Global Land Monitoring Group, Section 1.4 Remote Sensing and Geoinformatics, GFZ Helmholtz Centre for Geosciences, Potsdam, Germany; 4 Instituto de Pesquisa Ambiental da Amazônia (IPAM), Brasília, Brazil; 5 Universidade Federal do Maranhão (UFMA), São Luís, Brazil; 6 National Institute for Space Research (INPE), São José dos Campos, Brazil

**Keywords:** Forest ecology, Ecosystem ecology

## Abstract

Understanding the fate of carbon stocks in human-modified tropical landscapes is critical for mitigating climate change. Yet quantifying the impacts of landscape connectivity on the potential of regrowing forests to sequester carbon remains underrepresented. Using remote sensing and a space-for-time substitution approach, we analyzed aboveground carbon accumulation across the Brazilian Atlantic Forest. Forest connectivity emerged as a key determinant of carbon gains, with accumulation rates increasing by 43%–69% from fragmented to highly connected landscapes. In the western and coastline region, highly connected forests accumulated over three times more carbon (3.03 ± 0.81 vs. 0.93 ± 0.34 Mg C ha⁻¹ yr⁻¹) than those in low-connectivity areas. We modeled carbon stocks and found that full protection of secondary forests as of 2020 could increase stocks by 35% (132 Tg C) by 2030. Our results highlight the importance of protecting both old-growth and secondary forests while enhancing connectivity through targeted restoration. Strengthening conservation policies that integrate spatial connectivity is essential to maximizing the climate mitigation potential of tropical forests.

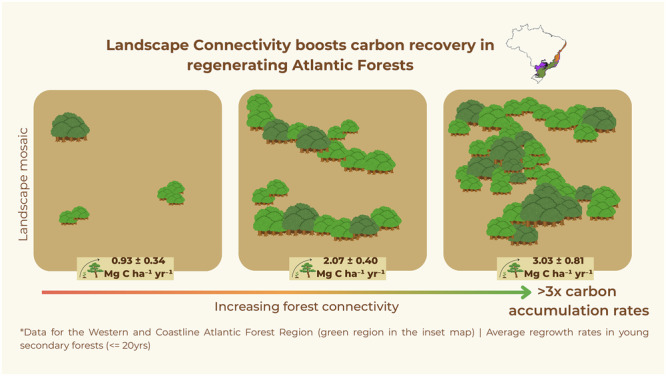

## Abstract (web summary)

Landscape connectivity strongly accelerates carbon recovery in Brazil’s Atlantic Forest, boosting accumulation rates by 43–69% and more than threefold in highly connected regions, based on remote sensing and a space-for-time substitution approach.

## Introduction

Forests play a pivotal role in regulating the Earth’s climate by safeguarding biodiversity and carbon stocks, and providing essential ecosystem services^1,2^. It is projected that global forests will account for approximately one-quarter of the pledged mitigation measures set out in the 2015 Paris Agreement^3^. This will be achieved through the control of deforestation and the promotion of forest regrowth. In this context, secondary forests—those regenerating after deforestation—are increasingly recognized for their key role in carbon sequestration, biodiversity conservation, and water security^2,4,5^.

A fundamental, yet unresolved, question is how carbon stocks in regrowing secondary forests behave in highly dynamic, human-modified tropical landscapes. While it is well established that forest fragmentation reduces carbon storage through edge effects^6,7^, we lack a detailed understanding of how landscape structure, especially forest connectivity, shapes the spatial variability of regrowth rates of secondary forests. This is a critical gap, as restoration initiatives and carbon crediting mechanisms often do not account for the landscape-level gains that can occur in adjacent forest remnants as connectivity improves and edge effects are buffered. Although previous studies have estimated broad aboveground carbon accumulation rates in secondary forests, they often overlook how landscape configuration influences these rates. Addressing this limitation could refine how restoration impacts are assessed and incentivized, considering the optimization of carbon accumulation across the landscape.

To address this gap, we use the Brazilian Atlantic Forest as a case study, which is a globally recognized biodiversity hotspot^8^ and a key region for tropical forest restoration^9,10^. This biome, as defined under Brazil’s national biome classification, originally occupied approximately 140 Mha along the eastern Brazilian Atlantic coast, expanding across heterogeneous latitudinal environmental conditions. However, it has been subject to significant human alteration since the sixteenth century, resulting in a considerable reduction of its original area to around 12%–24%, with critical landscape fragmentation^11^. Despite its protection laws, it continues to experience illegal old-growth forest loss, with secondary vegetation covering about 29% of recently deforested areas^12^.

Brazil has committed to restoring 12 million hectares (Mha) of forest by 2030 under its Nationally Determined Contribution (NDC) to the Paris Agreement. Within this context, the Atlantic Forest region stands out as a major restoration opportunity, and has been estimated to have between 21.6 and 36 Mha^13,14^ of land potentially available for regeneration. National and subnational programs, such as the Atlantic Forest Restoration Pact, have made notable progress toward these goals^15^. Yet the carbon accumulation trajectories of these regenerating landscapes, and the role of connectivity in shaping them, remain poorly quantified.

In this study, we integrate remote sensing data and a space-for-time substitution approach^16^ to quantify aboveground carbon accumulation rates in secondary forests across three distinct environmental regions of the Brazilian Atlantic Forest, which were grouped based on similarities in Maximum Cumulative Water Deficit (MCWD), average maximum temperature, and terrain slope. In this study, we explicitly assess how forest connectivity influences regrowth curves, intending to understand its role in carbon accumulation across landscapes. We then use the three regional regrowth models to estimate the carbon stocks of standing secondary forests as of 2020 and project future sequestration potential in 2030 and 2050.

By integrating spatially explicit data of landscape connectivity and regrowth models, our findings provide a more detailed understanding of the landscape-scale drivers of carbon accumulation in tropical secondary forests. This enables more accurate projections of regrowing forests’ carbon potential, supports the refinement of carbon crediting methodologies, and helps identify restoration strategies that optimize both ecological integrity and climate mitigation outcomes.

## Results

### Regional patterns and drivers of secondary forest regrowth in the Brazilian Atlantic Forest

**Fig. 1:**
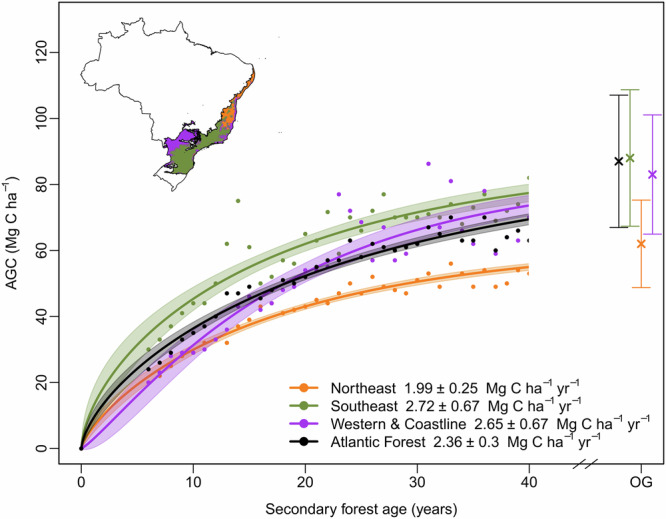
Atlantic secondary forest aboveground carbon accumulation with increasing age for each environmental region and for the whole Atlantic Forest. The environmental regions are grouped according to similarities in average annual maximum cumulative water deficit (MCWD; mm yr^−1^) between 1985–2020, average maximum temperature between 1985–2020 from the ERA5 dataset^52^, and terrain slope from the SRTM dataset^56^. Shading denotes the 95% confidence interval from the Chapman-Richards regrowth model^60^, based on the median value of the initial aboveground carbon for each age (dots in the figure). Crosses denote the median aboveground carbon of old-growth (OG) forests for each region (may include forests older than 35 years due to the data availability), and the uncertainty line represents the median and 95% CI. The legend shows the 20-year average growth rate of secondary forests in each region and the average confidence interval (±95% CI).

To model the regrowth rates of secondary forests across the Brazilian Atlantic Forest (BAF), we used samples from the MapBiomas annual land cover product (collection 7.1)^17^ to identify the secondary forest fragments and their respective ages from 1986 to 2020. These data were then associated with biomass values extracted from the European Space Agency Climate Change Initiative (ESA-CCI) Aboveground Biomass product for 2020 (version 4)^18^. The Brazilian Atlantic Forest has a strong latitudinal influence on its vegetation gradient. Therefore, the dataset was subdivided into three regions based on similarities in climatic and environmental variables to model region-specific regrowth curves (see Methods for detailed information on region clustering). The Northeast region has the most negative MCWD (most drought severe), an average maximum temperature of 25.5 °C, and a moderately inclined terrain. The Southeast region is on the lowest range of MCWD (less drought severe among the regions), the lowest average maximum temperature (22.7 °C), and the highest slope (steepest areas). Western and coastline regions are in the mid-range of MCWD (moderate drought), have the highest average maximum temperature (25.7 °C), and the terrain has the lowest average slope (Supplementary Table 1). Our results show significant differences in aboveground carbon (AGC) accumulation considering these three different regions and a baseline curve for the Atlantic Forest (Fig. 1). In the first 20 years of recovery, the secondary forests’ average annual growth rate in the Southeast region was 2.72 ± 0.67 (average±CI) Mg C ha^−1^ yr^−1^. This is 2.6% and 36.7% higher than the Western and Coastline region (2.65 ± 0.67 Mg C ha^−1^ yr^−1^) and the Northeast region (1.99 ± 0.25 Mg C ha^−1^ yr^−1^), respectively. Based on the estimated growth curves, secondary forests would require approximately 47, 62, and 52 years to reach the median aboveground carbon (AGC) of old-growth forests in the Western and Coastline, Southeast and Northeast regions, respectively, assuming that prevailing environmental conditions remain unchanged.

**Fig. 2:**
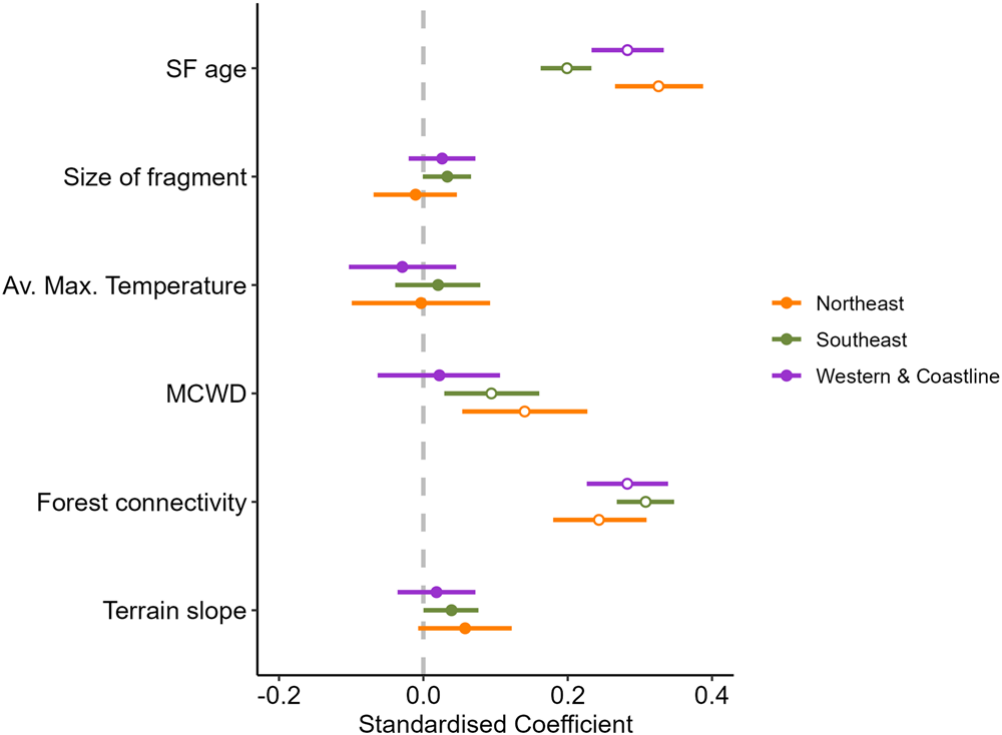
Standardized beta coefficients of the drivers of Atlantic secondary forest aboveground carbon stock for each environmental region. The values shown are the average standardized beta coefficients from GLS linear models runs considering the spatial autocorrelation of the samples (see “Methods” for detailed information). Bars represent the average standard deviation of the runs. White dots within the large circles represent whether the result was statistically significant (*p* < 0.05). The predictors and model outputs can be found in Supplementary table 2.

We employed a multilinear analysis to evaluate the impact of six environmental variables on aboveground carbon stocks in secondary forests across the three study regions. The predictor variables included secondary forest age, average maximum temperature, Maximum Cumulative Water Deficit (MCWD), terrain slope, fragment patch size, and forest connectivity. Despite the significant effect of MCWD in the Northeast and Southeast regions, landscape structure, especially connectivity have a greater impact on the regrowth across all the three regions of Atlantic Forest (*p* < 0.001) (Fig. 2). We hypothesize that the higher regrowth rates in the Southeast region are likely attributed to the fact that this region has a higher frequency of connected forest fragments (Supplementary Fig. 3) compared to the other regions and it is the region where forest connectivity had the strongest positive relationship with secondary forest carbon stocks (Fig. 2., *p* < 0.001).

### The influence of landscape connectivity on carbon accumulation

**Fig. 3:**
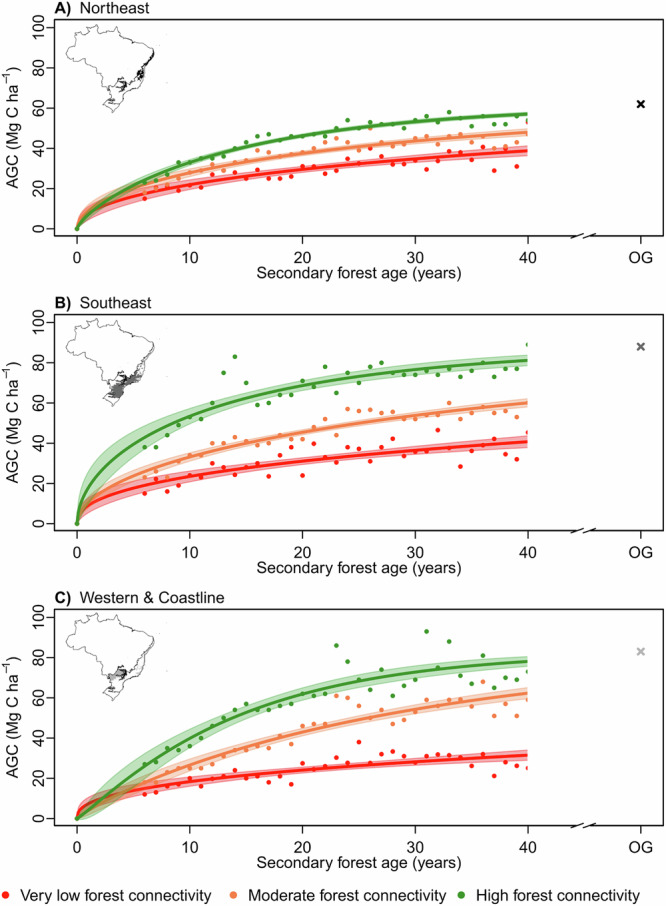
Atlantic secondary forest aboveground carbon accumulation with increasing age for each environmental region and forest connectivity categories. **A** Secondary forest carbon accumulation rates within forest connectivity categories for the Northeast region; **B** Secondary forest carbon accumulation rates within forest connectivity categories for the Southeast region; **C** Secondary forest carbon accumulation rates within forest connectivity categories for the Western and Coastline region. The forest connectivity categories are based on the Forest Area Density (FAD) algorithm. Shading denotes the 95% confidence interval from the Chapman–Richards regrowth model^60^, based on the median value of the initial aboveground carbon for each age and forest connectivity category (dots in the figure). Crosses denote the median aboveground carbon of old-growth (OG) forests for each region. The map within each plot shows the location of each region within the Atlantic Forest limits. The histogram distribution of samples in each connectivity category for each environmental region can be found in Supplementary Fig. 3.

Based on the relevance of landscape structure on the regrowth dynamics of secondary forests (Fig. 2), we investigate the isolated effect of connectivity across the three regions. We modeled the regrowth rates, categorizing landscapes based on their forest connectivity levels: very low (isolated fragments), moderate (patchy fragments), and high (fully connected forests) (details on the levels of connectivity are in the methods section). Our results reveal significant differences in carbon regrowth rates under varying connectivity conditions across all regions. Specifically, we found that the carbon accumulation rate in the first 20 years is reduced by 43%, 57%, and 69% when transitioning from high to very low forest connectivity landscapes in the Northeast (NE), Southeast (SE), and West & Coastline (W&C) regions, respectively (Fig. 3 and Supplementary Table 3). These differences exceed the 25–27% reduction observed across climatic-environmental gradients (Fig. 1), such as between a drier region (NE) and regions less affected by persistent droughts (SE, W&C), suggesting that landscape structure may play a critical role during early regrowth. For instance, in the W&C region, secondary forests within high-connectivity forest landscapes accumulate carbon at a rate more than three times greater than those in very low-connectivity areas (3.03 ± 0.81 Mg C ha^−^^1^ yr^−^^1^ vs. 0.93 ± 0.34 Mg C ha^−^^1^ yr^−^^1^, respectively; Fig. 3c and Supplementary Table 3).

The time required to reach 95% of the old-growth AGC stock varies considerably with forest connectivity. In the Northeast region, low-connectivity landscapes would take approximately 191 years, moderate-connectivity areas would require 98 years, and high-connectivity landscapes would take 47 years. In the Southeast region, low-connectivity areas would require more than 200 years, whereas moderate-connectivity landscapes would take 141 years, and high-connectivity forests would take 41 years. Similarly, in the West & Coastline region, low-connectivity forests would require more than 200 years to reach this threshold, moderate-connectivity areas would take 87 years, and high-connectivity landscapes 41 years.

### The carbon potential of Brazilian Atlantic secondary forests

Based on the relevance of secondary forests for climate mitigation and given the differences in the regrowth rates across the three regions, we modeled the aboveground carbon stocks of standing secondary forests in the Atlantic Forest biome in 2020. We then projected these stocks for 2030 and 2050 using the three regional regrowth models (Fig. 1) under two distinct scenarios, assuming constant environmental and climatic conditions and allowing only forest age to vary over time. Scenario 1 represented a protection approach in which all secondary forests present in 2020 are protected and allowed to mature until 2030 and 2050. However, this scenario does not consider the observed lack of permanence in secondary forests. Although deforestation of the Atlantic Forest in advanced stages of succession is legally permitted only in special conditions, such as public utility cases, the definition of successional stages and law enforcement remains a challenge^19^. In the Atlantic Forest, suppression of native vegetation in the initial stage of succession is allowed for any purpose. Previous studies have shown that roughly one-third of these forests are lost again within just eight years^20^. To reflect this reality and assess the policy implications of short-term forest loss, we developed Scenario 2. Scenario 2, by contrast, considered only secondary forests that were over eight years old as of 2020, allowing them to continue aging while assuming that forests aged eight years or younger in 2020 were cleared.

**Fig. 4:**
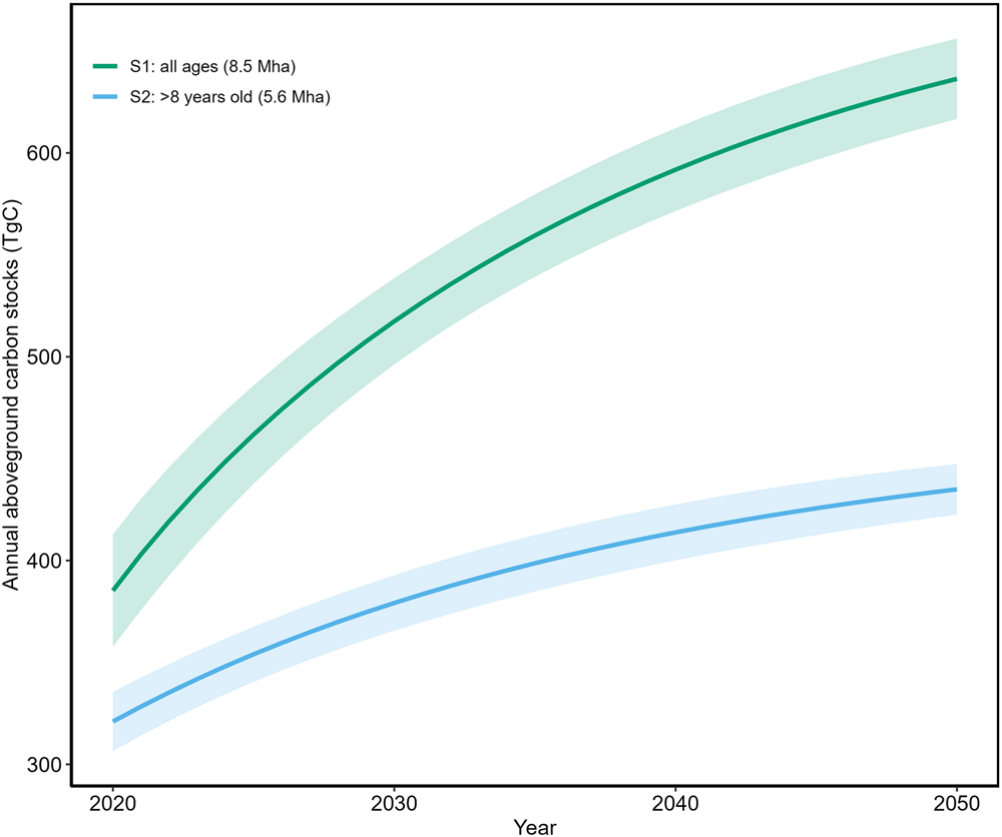
Aboveground carbon stock scenarios of secondary forests in the Atlantic Forest. The 2020 stock is based on the standing secondary forest area mapped from MapBiomas collection 7.1 masked with the Global Forest Watch plantation mask to exclude potential silviculture plantations, and then applied the respective regional regrowth curves to estimate the aboveground carbon stock. Scenario 1 assumes all the standing secondary forests are preserved until 2030 and 2050. Scenario 2 assumes only standing secondary forests older than 8 years in 2020 are preserved. The potential stock in 2030 and 2050 comprises the aged accumulated aboveground carbon stock from secondary forests in 2020. The stocks were calculated using the three region-specific regrowth models developed in this study.

**Fig. 5:**
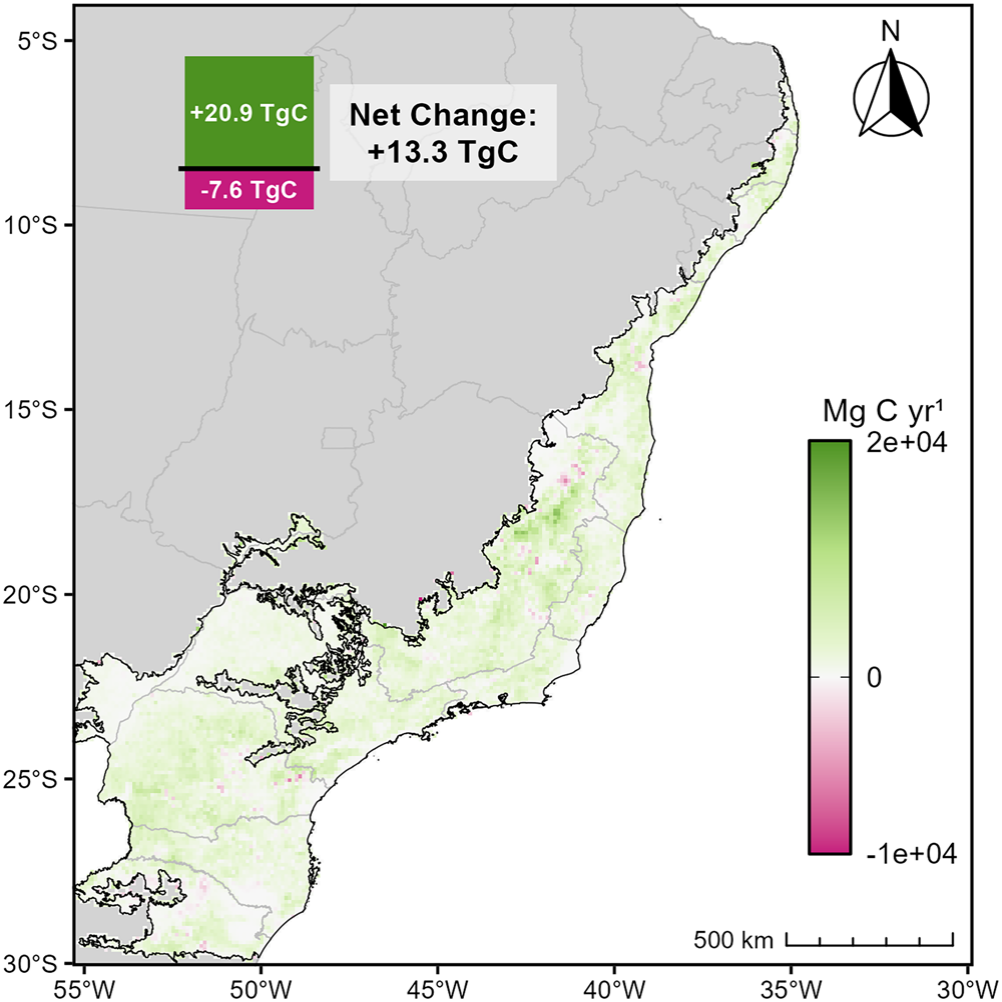
Spatial variation of the net aboveground carbon stock change of secondary forests between 2019 and 2020. Pink tones are the loss of carbon stocks due to secondary forest loss, and green tones are the gains of carbon stocks due to secondary forest recruitment and regrowth. All data is based on the standing secondary forest of the MapBiomas collection 7.1 processed data and the three regional regrowth models from this study.

Under Scenario 1 (Supplementary Table 4 and Fig. 4), the estimated carbon stock in 2020 was 385 (CI: 358, 413) Tg C across 8.49 million hectares (Mha) of secondary forests (Fig. 5). If these forests are preserved, the projected carbon stock would increase by approximately 35% to 517 (CI: 496, 539) Tg C by 2030 and 66% to 636 (CI: 617, 656) Tg C by 2050. In Scenario 2, the initial carbon stock in 2020 was estimated at 321 (CI: 307, 336) Tg C from secondary forests older than eight years. If these forests alone are preserved, the projected carbon stock would increase by approximately 18% to 379 (CI: 366, 393) Tg C by 2030 and by approximately 36% to 435 (CI: 423, 447) Tg C by 2050. Comparing scenario 2 to scenario 1 highlights the substantial impact of clearing younger secondary forests on long-term carbon sequestration. By 2030, projected carbon stocks under Scenarios 2 represent a reduction of approximately 27%, relative to Scenario 1. By 2050, this reduction increased to approximately 32%. These findings show the critical role of preserving all secondary forests in maximizing long-term carbon storage potential in the region.

We further estimated that, in 2020, gross carbon emissions from deforestation in old-growth forests in the Brazilian Atlantic Forest region were approximately 10.3 Tg C yr^−^^1^ (see “Methods” for details). Meanwhile, the net aboveground carbon sink of secondary forests between 2019 and 2020 reached 13.3 Tg C yr^−^^1^ (Fig. 5), which would offset these gross emissions from old-growth forest loss. This additional analysis, independent of the scenarios described above, was based on the standing secondary forests in 2019 and 2020 and provides a snapshot of short-term carbon stock changes in the biome.

## Discussion

### Regional variation in secondary forest carbon accumulation

Our study provides new insights into the dynamics of secondary forest regrowth across fragmented tropical forests, highlighting the major influence of landscape connectivity and, to a lower degree, regional environmental conditions on carbon accumulation. While we observed significant differences in AGC accumulation across the Northeast, Southeast, and West & Coastline regions, these regional patterns serve not as isolated findings but as evidence of the consequences of underlying drivers, such as water stress and landscape structure. The Northeast region, which experiences more persistent water deficits, exhibited the lowest AGC accumulation rates (1.99 ± 0.25 Mg C ha^−^^1^ yr^−^^1^), while the Southeast and Western & Coastline regions showed relatively higher rates (2.72 ± 0.67 Mg C ha^−^^1^ yr^−^^1^ and 2.65 ± 0.67 Mg C ha^−^^1^ yr^−^^1^, respectively). Moreover, we found that all regions had a stronger influence of forest connectivity in driving the aboveground carbon stocks of secondary forests than the water deficit. This shows the important role of landscape structure in boosting secondary forest recovery. These findings show the importance of regionally informed and process-based restoration strategies. Recognizing how climate and landscape configuration influence carbon dynamics allows for more targeted interventions, such as prioritizing restoration projects that benefit landscape connectivity.

### The role of landscape connectivity in enhancing forest regrowth

Beyond forest age, our analysis identifies forest connectivity as the strongest predictor of secondary forest carbon stocks across all regions in the Atlantic Forest. We also found that highly connected forest landscapes (here defined as the connectivity between old-growth and secondary forests) have older forests (Supplementary Fig. 6), which can benefit the stability of carbon stocks and reduce the associated environmental stresses and edge effects in both secondary and old-growth forests^21^. Our study shows that secondary forests in high-connectivity landscapes accumulate carbon up to three times faster than in low-connectivity areas, with secondary forests in low-connectivity requiring more than 200 years to reach 95% of the AGC levels of old-growth forests, compared to only ~41–47 years in high-connectivity landscapes. Previous studies have shown nearby forest cover as a main predictor of carbon stocks across Brazilian biomes^22^. The higher regrowth rates observed in greater forest landscape connectivity align with ecological mechanisms that facilitate forest recovery. This can be attributed to more effective seed dispersal from old-growth forests due to the role of larger, mobile taxa, such as mammals and birds, which facilitate seed dispersal and thereby promote the re-establishment of plant communities^23–25^. Furthermore, the availability of various propagules also underpins the higher regrowth rates observed in more connected forest landscapes, enriching the available seed pool, benefiting biodiversity and enhancing the capacity of secondary forests to recover and maintain their ecological functions over time^23,26^. Conversely, isolated secondary forest patches experience slower recovery rates due to limited seed dispersal and animal biodiversity loss^25^, harsher environmental conditions, and edge effects^7,27^ that reduce biomass accumulation and negatively impact secondary vegetation structure. Additionally, these more fragmented landscapes may have undergone earlier or more intense deforestation and associated disturbances, such as fire events, which could have long-lasting legacy effects on forest recovery, with fragmentation acting as a proxy for such historical impacts.

Our findings suggest that the trends observed in the Atlantic Forest, which is a highly fragmented landscape, could serve as a warning for other tropical forests facing increasing fragmentation, such as the Amazon^21,28,29^. Selective logging, deforestation, and expanding human activities are leading to growing fragmentation in tropical forests and increasing carbon losses due to edge effects^28,30–32^. As these forests become more fragmented, they may exhibit similar constraints on secondary forest recovery, with reduced carbon sequestration potential and longer recovery times in low-connectivity landscapes. Therefore, the Atlantic Forest serves as a potential future analogy for other forest ecosystems, showing the urgent need for conservation efforts that mitigate fragmentation and support connectivity-focused restoration strategies.

### Implications for restoration strategies

Our results emphasize the strong positive influence of forest connectivity on aboveground carbon accumulation in human-modified tropical landscapes. As secondary forests become more connected to older forest patches, they benefit from natural seed dispersal and improved microclimatic conditions that enhance seedling establishment and growth, and consequently, faster carbon accumulation. Moreover, increased secondary forests can reduce the fragmentation and buffering edge effects in old-growth forests, benefiting the stability of old-growth forest functions^21^. These findings highlight the critical need for conservation strategies that prioritize landscape connectivity to enhance forest recovery as well as co-benefits for old-growth forests. Restoration efforts should not only aim to expand forest cover but also strategically improve connectivity between forest patches to optimize carbon sequestration, with additional benefits for biodiversity recovery^4,33^ and potentially for other ecosystem services, such as pollination of crops. Therefore, employing a landscape approach to implement forest restoration would result in a dual climate mitigation impact, by increasing carbon sequestration and stability both at remnants and restored sites. Consequently, carbon crediting mechanisms should consider these dual impacts to financially support restoration interventions.

The observed low recovery rates in highly fragmented landscapes suggest that natural regeneration alone may be insufficient in these areas. Given the severe dispersal limitations and environmental stressors in such landscapes, active restoration strategies, such as direct seeding, assisted natural regeneration, and enrichment planting, may be necessary to accelerate forest recovery and ensure long-term resilience^34^. However, natural regeneration should be prioritized wherever feasible, regardless of landscape context, as it tends to be more cost-effective and ecologically efficient^2^. Even within fragmented landscapes, there are areas with potential for natural regeneration that should be identified and protected. In contrast, highly connected landscapes, where natural seed dispersal processes are less impacted, may benefit more from passive/natural restoration approaches that allow for spontaneous regeneration while maintaining ecosystem integrity. Therefore, restoration strategies should be context-dependent, taking into account both the degree of landscape connectivity and the ecological potential for regeneration. Strategic investments in ecological corridors and connectivity-focused restoration can further enhance outcomes, providing significant climate mitigation benefits while supporting forest ecosystem recovery and biodiversity conservation. Moreover, the long-term protection and management of regenerating areas are essential to ensure their transition into restored forests capable of delivering lasting environmental benefits^33^.

### The role of Brazilian Atlantic secondary forests in climate mitigation goals

Forest restoration can help Brazil to achieve its NDC goals of reducing net national emissions by 53.1% in 2030 compared to 2005 levels (698 Tg C yr^−^^1^), which is equivalent to an absolute net greenhouse gas emissions of 327.3 Tg C yr^−^^1^ in 2030. Here, we analyzed two preservation scenarios based on the standing forest of 2020. Under Scenario 1, we assume that no secondary forest present in 2020 is deforested and no new secondary forest is established. Under this scenario, carbon sequestration in secondary forests within the Atlantic Forest region could provide an additional ~3% (10 Tg C yr^−^^1^) reduction toward Brazil’s 2030 NDC target. Scenario 2, which considers only secondary forests older than eight years as of 2020, is more likely to persist and continue accumulating carbon according to the average turnover of secondary forests^20^. Under this assumption, the potential contribution drops to ~1.4% (4.5 Tg C yr^−^^1^), therefore highlighting the important contribution of protecting young secondary forests to optimize carbon sequestration^5^. Previous work using similar methods to model the regrowth rates focused on the Brazilian Amazon, estimated that preserving all standing secondary forests in that biome as of 2017 could contribute ~19 Tg C yr^−^^1^ toward Brazil’s 2030 NDC target^16^. When combined, the potential contributions from the Brazilian Amazon and Atlantic Forest could total up to ~8.8% (29 Tg C yr^−^^1^) of the emission reductions needed to meet Brazil’s current 2030 NDC goals. These findings highlight the significant role that protecting all secondary forest ages, as well as stopping deforestation and protecting all standing forests, can play in national climate mitigation strategies across Brazilian biomes.

We show that in 2020, the net aboveground carbon gains from secondary forests surpassed the carbon losses from old-growth forest loss. However, despite this positive sink, it is important to recognize that this apparent offset does not equate to broader ecological benefits. Old-growth forests have unique characteristics that may not be achieved by secondary forest recovery. For instance, old-growth forests serve as substantial stable carbon reservoirs, storing carbon accumulated over centuries in their above and belowground biomass and soils, and hold greater biodiversity. Continued loss of old-growth forests leads to further forest fragmentation, unprecedented biodiversity loss, and disruptions to ecosystem functions^35,36^. Although secondary forests exhibit higher rates of carbon sequestration, we show that they require longer periods to reach the total carbon storage capacity of old-growth forests, corroborated by previous studies^37^, and may take longer to achieve old-growth biodiversity^38^. Consequently, the decline of old-growth forests may jeopardize both the recovery of future secondary forests and the success of forest regeneration projects and the NDC goals.

### Regrowth rates in the context of prior work

When compared to previous large-scale estimates for the Atlantic Forest, our study highlights important regional and landscape-scale variations in secondary forest regrowth (Supplementary Fig. 11). Field-data integration studies and remote sensing studies, respectively, suggest an average regrowth rate of 3.59 Mg C ha^−^^1^ yr^−^^1^ (Cook-Patton et al.), 2.61 Mg C ha^−^^1^ yr^−^^1^ (IPCC, 2019), and 2.57 Mg C ha^−^^1^ yr^−^^1^ (Chen et al.) across the entire biome. Our study estimated a 2.36 Mg C ha^−^^1^ yr^−^^1^ for the entire biome. In contrast, the National Greenhouse Gas Inventory (NGHGI) from Brazil reports a significantly lower estimate of 1.66 Mg C ha^−^^1^ yr^−^^1^ for the Atlantic Forest. Our region-specific findings reveal lower regrowth rates in the Northeast (1.99 Mg C ha^−^^1^ yr^−^^1^), with only Western & Coastline (2.65 Mg C ha^−^^1^ yr^−^^1^) and the Southeast regions (2.72 Mg C ha^−^^1^ yr^−^^1^) approaching the lower bound of previous studies for the entire biome, but still higher than the estimate of the NGHGI. These discrepancies highlight the importance of accounting for regional environmental conditions, as the Atlantic Forest is highly heterogeneous in climate, topography, and historical land use. The NGHGI estimate is about 15–54% lower than our regional estimates and aligns more closely with our findings for secondary forests located in moderate to lower landscape connectivity. Incorporating forest connectivity as a key driver of secondary forest regrowth, our results demonstrate substantial variation within each region. These findings suggest that estimates based on a limited number of field plots may overestimate carbon accumulation in highly fragmented landscapes while underestimating the potential of well-connected secondary forests to sequester carbon at rates approaching or even exceeding biome-wide averages. Consequently, estimates from NGHGI and IPCC methodologies are unable to fully capture the spatial complexity of human-modified landscapes and the full geographical variability of these biomes. Therefore, remote sensing-based estimates bring a complementary perspective that better captures this spatial variability.

While this study provides valuable insights into the carbon accumulation estimation of secondary forests based on remote sensing data, limitations should be acknowledged. We show that the time to recover to similar AGC levels of old-growth forests is lower than what is reported for the Amazonian forest^16,39,40^. This might be a result of a lower old-growth forest AGC estimate for the Atlantic Forest. Specifically, because these old-growth forests might have suffered disturbances along their existence that are not accounted for here due to data limitations before 1985. A known limitation of remote sensing-derived biomass estimates is biomass saturation in high biomass forests and overestimation in low biomass regions^41^, which can be correlated with younger secondary forests. This overestimation may be attributed to the lower sensitivity of remote sensing-derived biomass models, which distinguish fine-scale variations in regrowth stages, leading to inflated biomass estimates for recently regenerating forests and resulting in lower growth rates. Another challenge arises from the influence of nearby land cover in highly fragmented landscapes. Small forest fragments, which are common in highly human-modified landscapes, tend to have greater influence of edge effects, which may not be captured by the spatial resolution of moderate-resolution datasets. Additionally, while we selected fragments ≥1 ha to roughly match the 100 m ESA CCI biomass pixels, some elongated or irregularly shaped fragments may not perfectly align with the pixel grid. However, using the median aboveground carbon from thousands of fragments helps mitigate potential biases due to fragment shape, pixel misalignment and saturation effect from the biomass data^42^. Moreover, the estimation of secondary forest age derived from MapBiomas land cover changes is subject to inherent uncertainties. While MapBiomas provides a valuable time-series dataset for tracking land use changes, its classification approach may not always accurately capture the exact establishment year of secondary forests and detect small-scale restoration areas^43^. To mitigate some of these limitations, we corrected the secondary forest age and its associated aboveground carbon from ESA CCI Biomass data using field data observations. Based on this analysis, we found that when the MapBiomas-based algorithm starts to detect a pixel as secondary forest, this forest has an equivalent aboveground carbon of a 5–6-year-old secondary forest (Supplementary Fig. 5). We then used this to adjust the age and associated AGC in this study (see “Methods” section for detailed information). Future studies should explore the integration of higher-resolution remote sensing datasets and active remote sensing techniques, such as LiDAR and new missions (e.g., BIOMASS, NISAR). For instance, BIOMASS is a P-Band sensor and therefore might help to reduce potential saturation effect in high-biomass tropical regions^42^, however, old-growth baselines are needed to help build these recovery curves. Other missions, such as NASA-ISRO Synthetic Aperture Radar (NISAR), might also prove helpful to further improve aboveground biomass estimates as they have higher resolution. Therefore, refining land cover classification algorithms, improving biomass estimates derived from earth observation data, and incorporating field validation efforts will be essential for enabling such data to be used effectively by end-users such as restoration monitoring communities^44^.

## Conclusion

While previous biome-scale estimates provide valuable general benchmarks, our study shows the need for incorporating landscape connectivity and regional environmental differences when estimating the carbon sequestration potential of secondary forests. These insights reinforce the role of connectivity-enhancing restoration strategies, which are not only important for increasing carbon accumulation but also for promoting biodiversity and ecosystem resilience^45^ in fragmented tropical forests under a changing climate. Furthermore, our findings emphasize that young secondary forests should not be dismissed but recognized as vital carbon sinks. If properly conserved and integrated into climate policies, these forests can contribute significantly to long-term carbon sequestration. To this end, conservation strategies should prioritize the protection of both old-growth and secondary forests while promoting the restoration of degraded areas^5^. Such approaches can maximize carbon capture potential and simultaneously support biodiversity and the provision of critical ecosystem services. Overall, our study identifies two key priorities for advancing climate and conservation goals: (1) restoring forest connectivity to optimize carbon sequestration and co-benefits, and (2) addressing the pervasive increase in forest fragmentation. By embedding landscape connectivity into restoration planning, policymakers can significantly enhance the ecological and climate benefits of secondary forest recovery, thereby supporting national and global environmental commitments, particularly in highly fragmented regions.

## Methods

### Data preparation and sampling

To determine the secondary forest extent and age across the Atlantic Forest from 1986 to 2021, we employed the same methodology as previous studies^16,46^. This method utilizes land use and land cover maps to track conversions from anthropogenic land to forest land through a pixel-by-pixel analysis. Then, by counting the consecutive years following the transitions, as well as considering repeated deforestation, this method estimates the secondary forest age (in years). In this study, we updated the implemented algorithm in the Google Earth Engine platform with the land-use and land-cover map of MapBiomas (collection 7.1). The 2020 standing secondary forest age map, with ages ranging from 1 to 35 years, served as our baseline. Since our paper aimed to analyze natural secondary forest regrowth, a forest plantation mask from the Global Forest Watch^47^ was applied over the 2020 secondary forest age map to remove potentially misclassified pixels of forest plantations (e.g., silviculture of Pinus, Eucalyptus, etc) in the MapBiomas dataset. The remaining pixels of secondary forest age were grouped by age and location using the ‘RegionGroup’ function from ArcPy and converted to a vector format. Then, only the secondary forest fragments that were greater than 1 ha were selected for further analysis to match the spatial resolution of the aboveground biomass product (100 m). This vector file of secondary forest fragments using the projection system WGS 84, was used to extract all the zonal statistics we needed in the further analysis explained in the sections below. To produce an old-growth forest map, we assumed that forest land pixels that did not undergo any transition between 1985 and 2020 were old-growth in 2020. A limitation of our old-growth forest map is that secondary forests, which were deforested and regrew before 1985, were assumed to be old-growth. This limitation in our study is due to the temporal data availability of MapBiomas (1985–2021; collection 7.1). Moreover, biomass estimates for old-growth forests may be affected by unaccounted historical disturbances, potentially leading to underestimations. As the Atlantic Forest has been subject to human interventions for centuries, even old-growth fragments may not be as structurally intact or as old as those in less disturbed biomes, such as the Amazon. Similar to the secondary forest processing, the old-growth forest fragments were grouped and converted to a vector format. Then, we applied the same filtering to select only fragments greater than 1 ha, to avoid a mismatch with the spatial resolution of the aboveground biomass product. To extract the associated aboveground carbon (AGC) of the standing secondary and old-growth forests in 2020, we used the ESA-CCI Aboveground Biomass v4 (AGB)^18^ product (100 m of spatial resolution) for the year 2020. We converted this dataset to AGC by applying the conversion factor of 0.47, which is the biomass-to-carbon ratio used by the IPCC^48^. Both secondary and old-growth forest maps were laid over the AGC map, and key statistics (mean, mode, median, maximum, minimum and standard deviation) of AGC were calculated and extracted for each polygon of both secondary forest and old-growth forest using a zonal statistics function available in ArcPy. A boxplot showing the distribution of AGC for each secondary forest age can be found in Supplementary Fig. 7. The Atlantic forest is highly fragmented, and to avoid the high influence of the neighborhood on the old-growth forest AGC values, which we assume as the asymptote in our growth model (i.e., the maximum AGC potential), we selected old-growth fragments where the AGC was equal to or greater than 47 Mg C ha^−1^. This is equivalent to an aboveground biomass of 100 Mg ha^−1^, which is the average biomass value for old-growth Atlantic forests based on literature^49,50^. After the data filtering, we were left with a sample of 431,240 polygons of standing secondary forest fragments and 590,790 polygons of standing old-growth forest fragments in 2020 for further analysis.

### Secondary forest age and associated AGC correction and AGC validation against field data

To classify the land cover, MapBiomas methodology uses two temporal filters to confirm the classification of a pixel as a forest using a 3-year moving window to correct the classification of a class in the middle year and return to the same class in the next year. It then applies a moving window of 4–5 years to correct all middle years^17^. This is a conservative method to avoid misclassified transitions due to the classification methodology; however, it can make it difficult to map early-stage forest regeneration.

To support and validate the remote sensing-based estimates, we compiled a field database of aboveground carbon (AGC) derived from forest inventory data collected from 137 plots distributed across the Atlantic Forest biome between 2019 and 2024 as part of the project “Understanding restored forests for benefiting people and nature (NewFor)”. Each plot covered an area of 30 × 30 m, where all trees with a diameter at breast height (DBH) greater than 5 cm were measured. For each individual, DBH and total height were recorded, and species were identified to the species level when possible, or otherwise to the genus level, to allow assignment of woody density values from the Global Wood Density Database^51^. Aboveground biomass was estimated using allometric equations and converted to AGC assuming a carbon fraction of 0.47.

We calculated the median AGC from the ESA CCI map for each secondary forest age using the 431,240 polygons we sampled from Mapbiomas. We then used a non-parametric Wilcoxon signed-rank test to test whether the age range and associated median AGC from field data were not statistically significantly different from the median AGCage1 based on remote sensing data. For this analysis, we grouped the field data AGC into age groups of 2 to obtain a minimum number of samples for statistical analysis. We found that the median AGC of 5–6 year old secondary forests from field data was not statistically significantly different from the median AGC_age1_ from remote sensing (Supplementary Fig. 10). Based on this analysis, we then assumed that the corresponding median AGC_age1_ based on remote sensing was equivalent to the median AGC of a 6 year old secondary forest and used this to shift the median AGC and associated age from remote sensing to start at age 6 instead of age 1, and assumed that AGC in age 0 would be 0 Mg/C so that the growth model would start at 0.

### Environmental variables and regions definition

The climate variables (precipitation and temperature) used in our research were based on the ERA5 (1985–2020) dataset^52^. The ERA5 data was processed on the Google Earth Engine platform, where we calculated the average precipitation, average temperature and average maximum temperature between 1985 and 2020 for the Brazilian Atlantic Forest boundaries. The Maximum Cumulative Water Deficit (MCWD) is defined as the most negative value of climatological water deficit (CWD) over a year and was calculated using a script in R language based on the calculation of previous studies^53,54^. MCWD was calculated based on spatially explicit monthly ERA5 precipitation (*P*) data and spatially explicit monthly evapotranspiration (ET) from TerraClimate^55^ (1985–2020). Rather than assuming a constant evapotranspiration value, we used the spatially explicit evapotranspiration to better represent climatic heterogeneity across the Atlantic Forest. The CWD and MCWD was calculated as (Eq. 1); where *n* is month:

1 $${CWD}n	 = {CWD}n-1+{Pn}-{ET}{n;}\,{Max}\left({CWD}n\right)=0;\\ {{CWD}}_{0} 	= {{CWD}}_{12}{;}\,{MCWD} ={Min}({{CWD}}_{1}\,...{{CWD}}_{12})$$

We then calculated the average MCWD for the whole period (1985–2020) for use in subsequent analysis. The terrain elevation and slope data were based on the Shuttle Radar Topography Mission (SRTM) digital elevation dataset^56^. It is important to note that the ERA-5-derived climate layers (e.g., temperature, MCWD) and other environmental variables, such as elevation and slope, were resampled in Google Earth Engine to a 100 m grid using the default nearest-neighbor resampling method to overlay the secondary forest fragments layer for zonal statistics calculation. As such, the resulting fine-resolution raster retains the original coarse-scale values of climate data and does not reconstruct sub-pixel variability. This limitation means that fine-scale climatic heterogeneity within the original coarse pixel footprint may not be captured.

To separate the Atlantic Forest biome into three main regions by their environmental similarities, we used a machine learning unsupervised clustering method called the K-means algorithm. This clustering method groups similar data together without predefined labels. To determine the input variables for clustering the pixels based on the relationship with the AGC, we first calculated a correlogram to analyze collinear relationships between the environmental variables using a rank-based Spearman correlogram (Supplementary Fig. 8). Variables with correlations exceeding ±0.5 were excluded to minimize multicollinearity. The final set of predictors for the k-means clustering included mean maximum temperature, mean maximum cumulative water deficit (MCWD), and terrain slope. The number of clusters was set to three to capture the dominant bioclimatic gradients of the Atlantic Forest. This choice reflects the broad environmental heterogeneity of the biome, which spans cooler and wetter southern and high elevation areas, hotter and flatter western and coastal lowlands, and hotter and drier conditions in the northeast. Clustering solutions with a higher number of clusters (*k* = 4 and *k* = 5) primarily subdivided these broad regions without revealing clearly distinct climatic domains (Supplementary Fig. 9). The resulting three regions were consistent with major functional and bioclimatic divisions of the Atlantic Forest reported in previous studies^57,58^. The k-means clustering classification was performed on the Google Earth Engine platform.

### Landscape fragmentation/connectivity metric

To assess landscape fragmentation, we used the Forest Area Density (FAD) index, which quantifies the proportion of forest cover within a given neighborhood and is the recommended Food and Agriculture Organization (FAO) indicator for assessing forest connectivity at the landscape level. This metric provides a spatially explicit measure of forest connectivity by assessing the density of forest pixels within a moving window, effectively distinguishing core forest areas from more fragmented landscapes by classifying each forested pixel between 0 and 100. Higher FAD values indicate greater forest continuity, while lower values reflect increased fragmentation and forest isolation. A forest mask containing both secondary and primary forests in the year 2020 at 30 m of spatial resolution was derived from the MapBiomas 7.1 collection and used as input to calculate the FAD index. This analysis was performed all over Brazil using the Guidos Toolbox Workbench (GWB)^59^, accessible through the FAO SEPAL cloud computing platform. We used a moving window of 35 × 35 pixels (~1 km^2^) to calculate the FAD values. This window size was chosen to capture landscape-scale patterns of connectivity structure in an already highly fragmented landscape such as the Atlantic Forest. We then cropped the FAD output to the boundaries of the Atlantic Forest biome and extracted the median FAD value for each standing secondary forest fragment in 2020 using a zonal statistics approach. Then we subdivided it into high forest connectivity (FAD ≥ 40 and ≤100); moderate forest connectivity (FAD ≥ 10 and <40), and very low forest connectivity (FAD < 10).

### Secondary forest carbon accumulation

We used a space-for-time substitution approach to model AGC accumulation with increasing secondary forest age, following the method from previous work for the Amazon^16,40^. The AGC accumulation was modeled using the Chapman-Richards growth model^60^ (Eq. 2) in each of the three specific regions for the Atlantic forest.

2 $${Y}_{t}=A{\left({1-e}^{-{kt}}\right)}^{c}\pm {{\varepsilon;}}\,A,\,K\,{and}\,c > 0$$

In the Chapman–Richards growth model, the Yt refers to the AGC at each secondary forest age (*t*); *A* is the AGC asymptote, here defined as the AGC of old-growth forest; *k* is the growth rate coefficient of *Y* as a function of age; *c* is a coefficient that determines the shape of the growth curve, and *Ɛ* is the error term of the equation. We assumed that the secondary forest AGC could return to the equivalent old-growth forest AGC after a given number of years, therefore reaching a precalculated asymptote. We applied the Chapman–Richards model using the corrected associated median AGC of the sampled data of secondary forest by age (Yt term) and old-growth forest (A term). We used the median aboveground carbon from thousands of fragments to reduce the potential biases due to fragment shape, pixel misalignment and saturation effect from the biomass data^42^. Finally, we modeled the forest carbon accumulation for each of the three environmental regions of the Atlantic forest, as well as for each forest connectivity category (high forest connectivity, moderate forest connectivity and very low forest connectivity) in each of the three regions. We then compared our estimates of growth rates with estimates from previous studies^22,61^, the IPCC default rates—using the average of two ecozones (tropical rainforest and tropical moist forest) to match the Atlantic Forest^48^, and the National Greenhouse Gas Inventory (NGHGI) rates^62^.

### Importance of each driver

We used a Generalized Least Squares (GLS) with a bootstrapping approach to assess the relative importance of each independent variable in influencing the aboveground carbon (AGC) of secondary forests for each region. To minimize the effects of spatial autocorrelation, we fitted GLS linear models with all the spatial correlation structures available and selected the model with the lowest Akaike Information Criterion (AIC). Based on AIC, the linear model with an exponential spatial correlation structure was considered the best-fitting model (lowest AIC). This analysis was performed using the nlme R package^63^.

We then applied the GLS model with an exponential spatial correlation structure to determine the standardized coefficients of each environmental variable and the age of secondary forests in each region of the Brazilian Atlantic Forest. We ran the GLS model analysis 50 times for each region, randomly sampling 1% of the total samples of each region in each iteration. Following this, we calculated the average standardized beta coefficient, standard error, and *p*-value at the 95% confidence interval for each model predictor across all the iterations for the three regions.

### Potential carbon stocks scenarios

To calculate the potential carbon stock scenarios, we used 2020 as the baseline year for the standing secondary forests. To estimate the standing secondary forest ages in 2020, we used the MapBiomas collection 7.1 and masked it with the GFW plantation mask to exclude potential silviculture plantations. To estimate the secondary forest ages in 2030 and 2050, we first aged the 2020 standing secondary forests map by adding 10 and 30 years to their age in 2020, respectively. We then used the three region-specific modeled regrowth curves to estimate the total carbon stocks of available standing secondary forests for two different scenarios. Scenario 1 (S1) is the protection scenario, where all secondary forest ages remain standing and preserved; therefore, this represents the full carbon accumulation potential of the standing forests in 2020. In Scenario 2 (S2), we allowed only forests with an age older than 8 years in 2020 to be preserved; therefore, we assumed that all forests with an age equal to or below 8 years would be deforested in 2020.

### Estimation of carbon emissions from deforestation

Carbon emissions from old-growth deforestation in 2020 were estimated in this study using the deforestation area data available on the Terrabrasilis platform for the Atlantic Forest (PRODES Mata Atlantica). We used the Greenhouse Gas emissions and Removals Estimation System (SEEG)^64^ and the National Greenhouse Gases Inventory (NGHGI) as the reference for the carbon stock in old-growth forest. To estimate the emissions from old-growth forest loss, we multiplied the deforestation area in 2020 (79,070 ha) by the carbon stock in mature forest used by SEEG/NGHGI (130.38 tC ha^−1^).

## Supplementary information

Transparent Peer Review File

Supplementary Material

## Data Availability

All datasets supporting this study are publicly available on Zenodo (10.5281/zenodo.16838291). External data sources include the ESA CCI Biomass 2020 dataset (https://catalogue.ceda.ac.uk/uuid/af60720c1e404a9e9d2c145d2b2ead4e/), the MapBiomas dataset (https://brasil.mapbiomas.org/en/colecoes-mapbiomas/), and the TerraClimate evapotranspiration dataset (https://www.climatologylab.org/terraclimate.html). Code to generate secondary forest age from MapBiomas Collection 7 was written by Viola Heinrich. Code to calculate MCWD is available at https://github.com/celsohlsj/RasterMCWD.

## Code availability

All code supporting this study analysis and figures are publicly available on Zenodo (10.5281/zenodo.16838291).
