# Supplementary material for: Forest connectivity boosts carbon recovery in regenerating Atlantic Forests
Source: Commun Earth Environ. 2026 Apr 9;7(1):627. doi: 10.1038/s43247-026-03480-5 (PMC13427630; doi:10.1038/s43247-026-03480-5)
Supplement: Supplementary file 2 — Supplementary Material [file 43247_2026_3480_MOESM2_ESM.pdf]

Supplementary Figures and Tables

Forest connectivity boosts carbon recovery in regenerating Atlantic forests

Thais M. Rosan[1], Laura B. Vedovato[2], Viola Heinrich[3], Celso H. L. Silva-Junior[4,5], Pedro H. S. Brancalion[2], Stephen Sitch[1], Luiz E. O. C. Aragão[1,6]

1. Faculty of Environment, Science and Economy, University of Exeter, Exeter, United Kingdom
2. Department of Forest Sciences, “Luiz de Queiroz” College of Agriculture, University of São Paulo, Brazil
3. Global Land Monitoring Group, Section 1.4 Remote Sensing and Geoinformatics, GFZ Helmholtz Centre for Geosciences, Potsdam, Germany
4. Instituto de Pesquisa Ambiental da Amazônia (IPAM), Brasília, DF, Brazil
5. Universidade Federal do Maranhão (UFMA), São Luís, MA, Brazil.
6. National Institute for Space Research (INPE), São José dos Campos, Brazil.

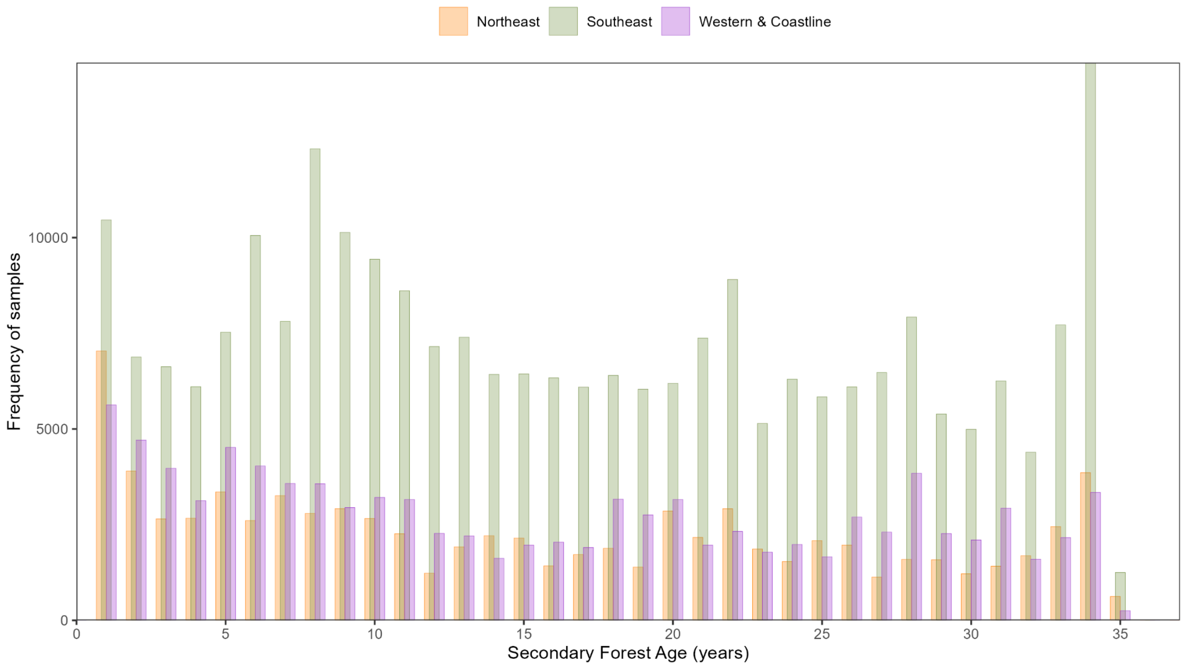

Supplementary Figure 1. Histogram with the sample distribution of secondary forest age in each environmental region of the Atlantic Forest.

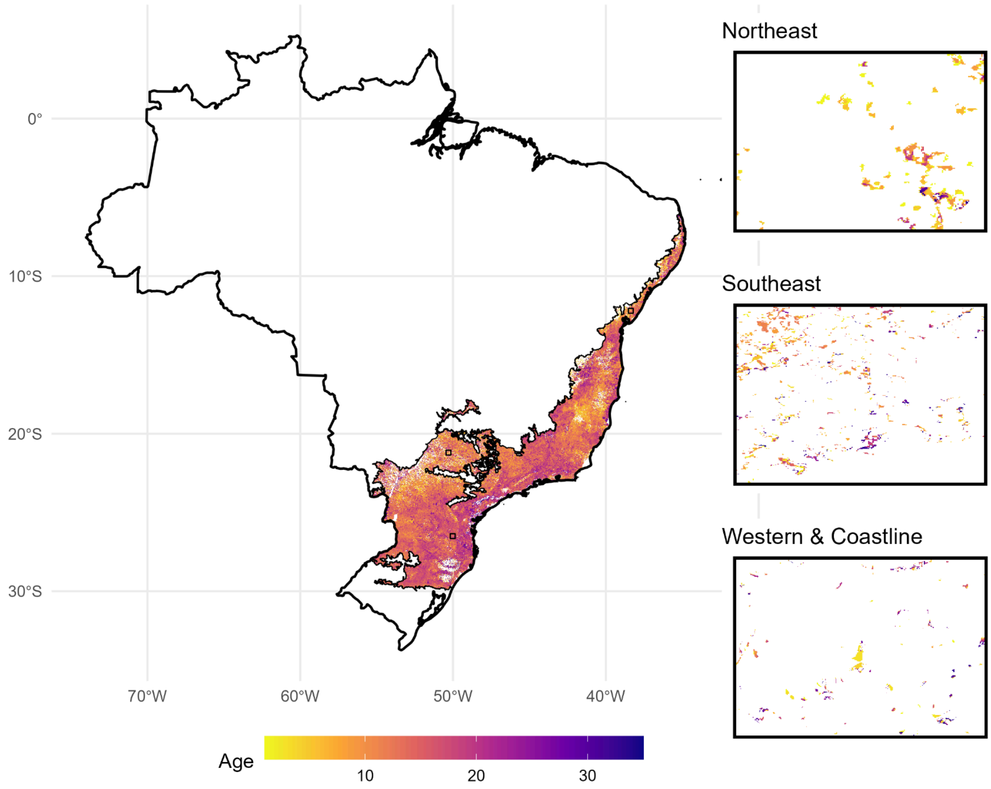

Supplementary Figure 2. Map showing the average/mean secondary forest age in the Atlantic Forest aggregated to a 1.5km^2 spatial. The inset maps show the original spatial resolution (30m) of the secondary forest age fragments for each region of Atlantic Forest defined in this study.

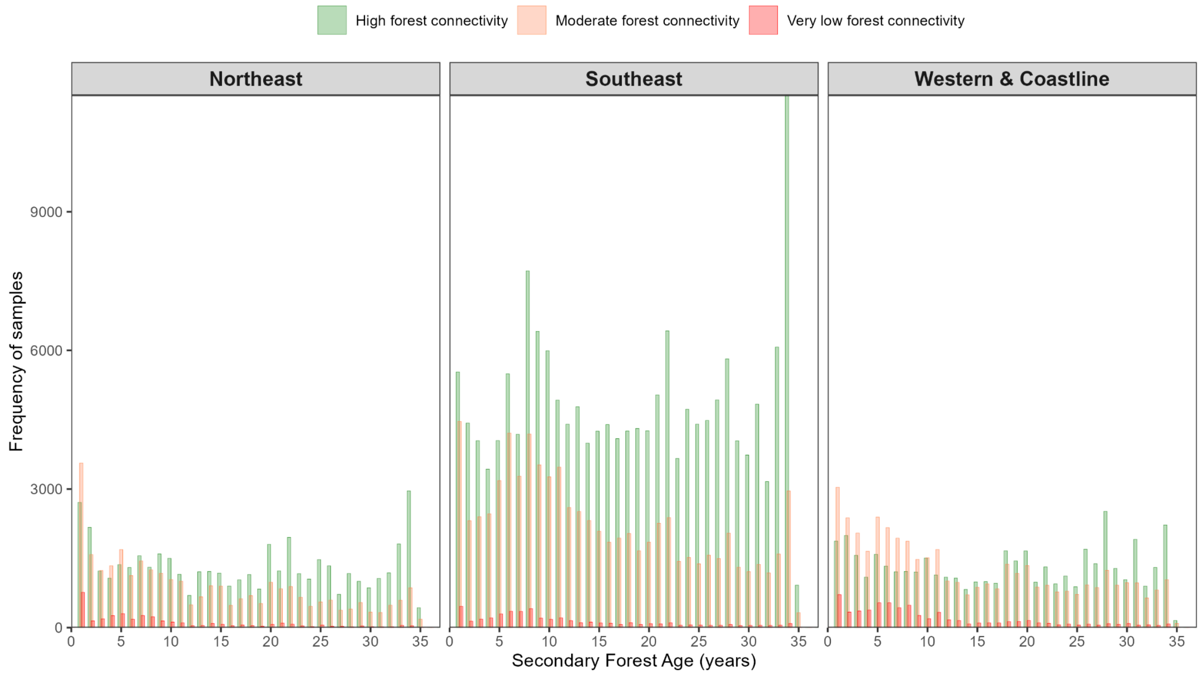

Supplementary Figure 3. Histogram with the sample distribution of forest connectivity categories by secondary forest age in each environmental region of the Atlantic Forest.

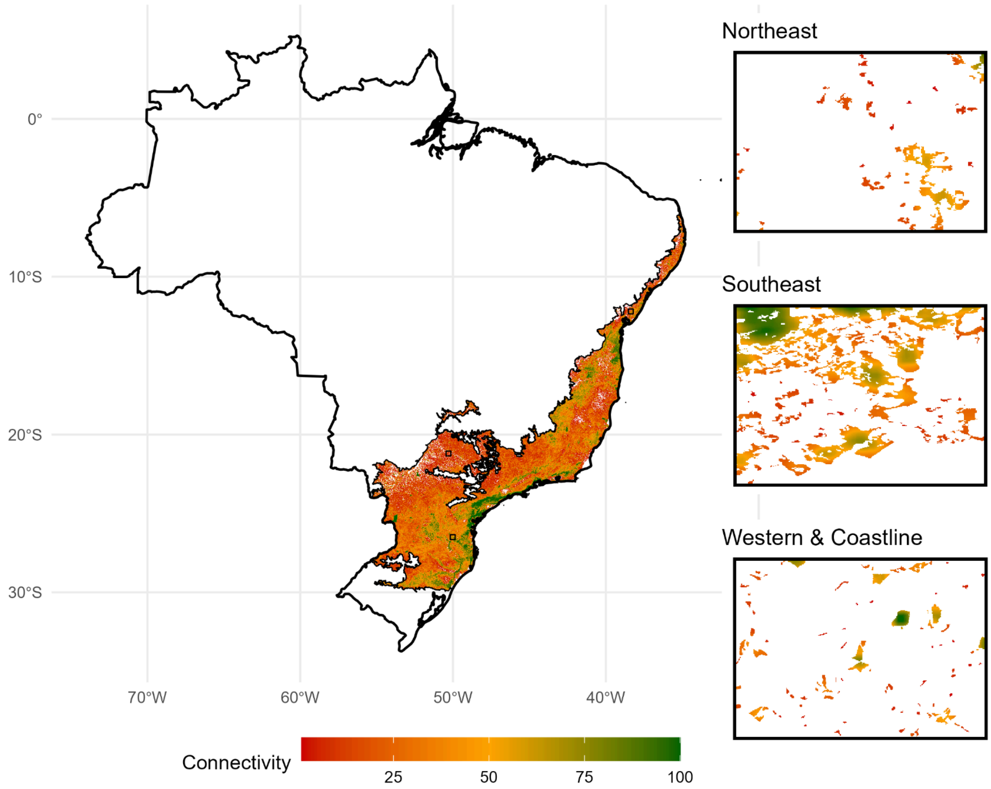

Supplementary Figure 4. Map showing the average FAD connectivity metric in the Atlantic Forest aggregated to a 1.5km^2 spatial resolution. The inset maps show the original spatial resolution (30m) of FAD for each region of Atlantic Forest defined in this study.

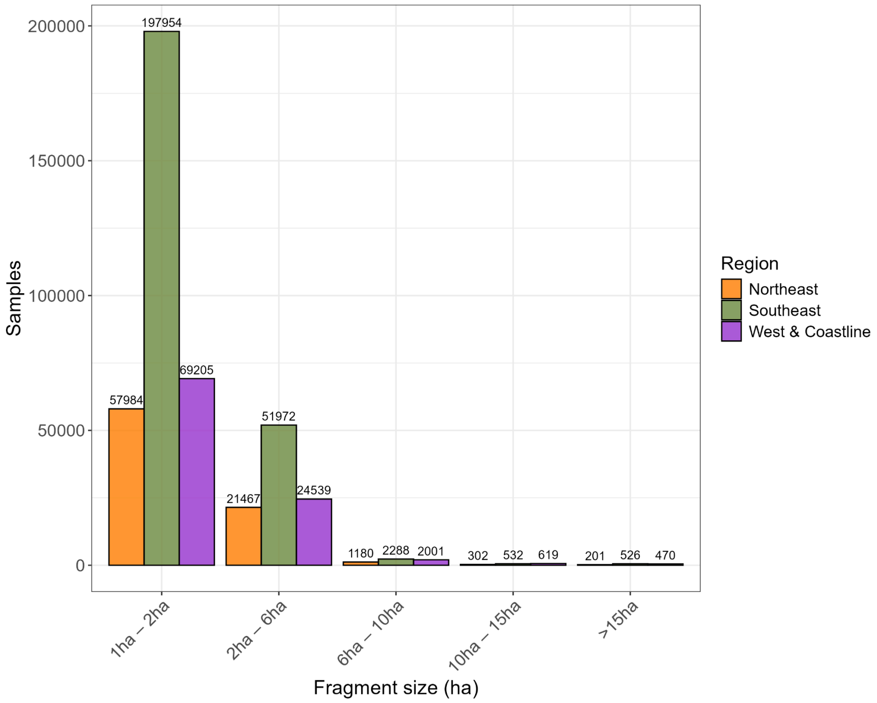

Supplementary Figure 5. Histogram with the sample distribution of the size of secondary forest fragments (ha) in each environmental region of the Atlantic Forest.

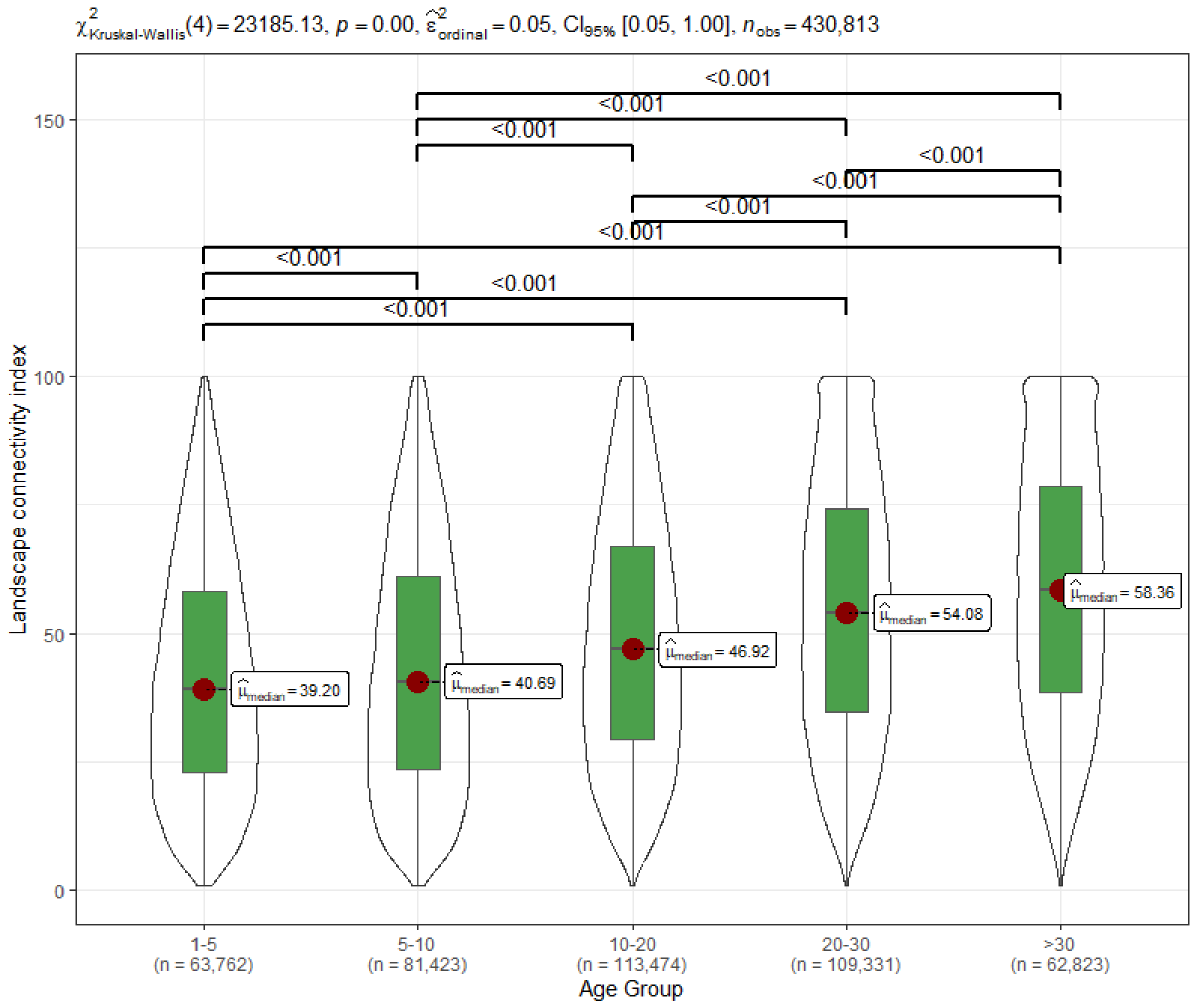

Supplementary Figure 6. Boxplot with the forest connectivity index distribution by secondary forest age groups.

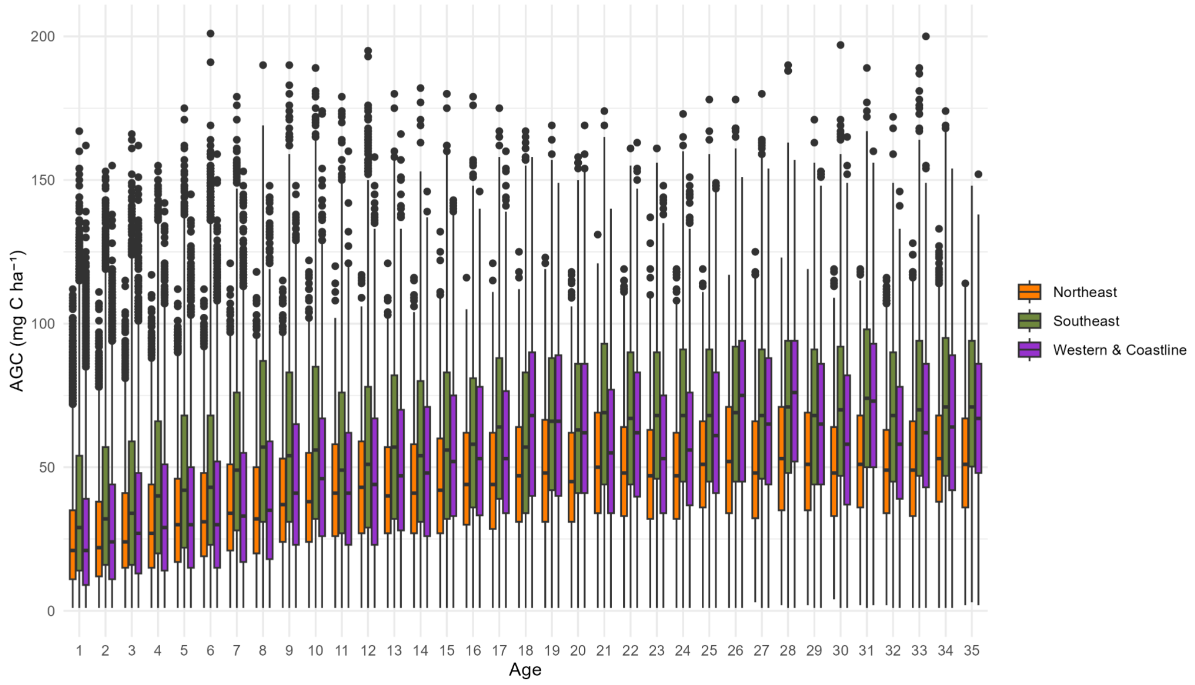

Supplementary Figure 7. Boxplot with the Aboveground Carbon (AGC) distribution by secondary forest age groups and Atlantic Forest regions.

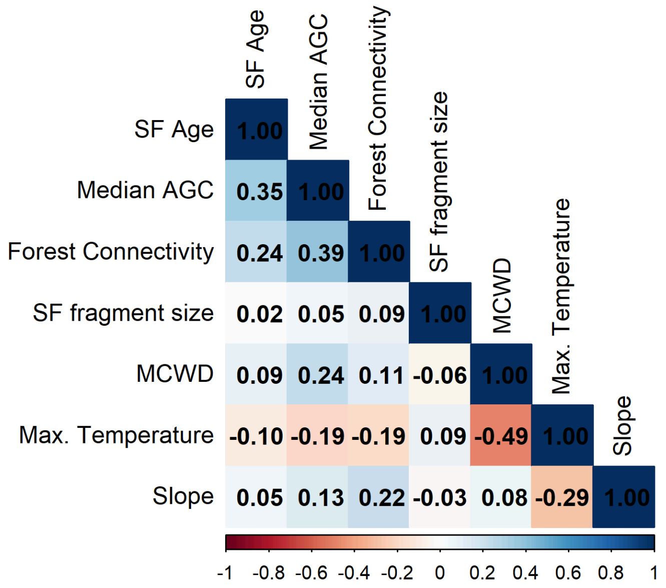

Supplementary Figure 8. Correlogram showing the ranked-based Spearman between the environmental variables and secondary forest age. The values within each box show Spearman's R value, and the colour shows the relative degree and sign of the relationship between the variables. Variables with no shading were non-significant at the 95% confidence interval.

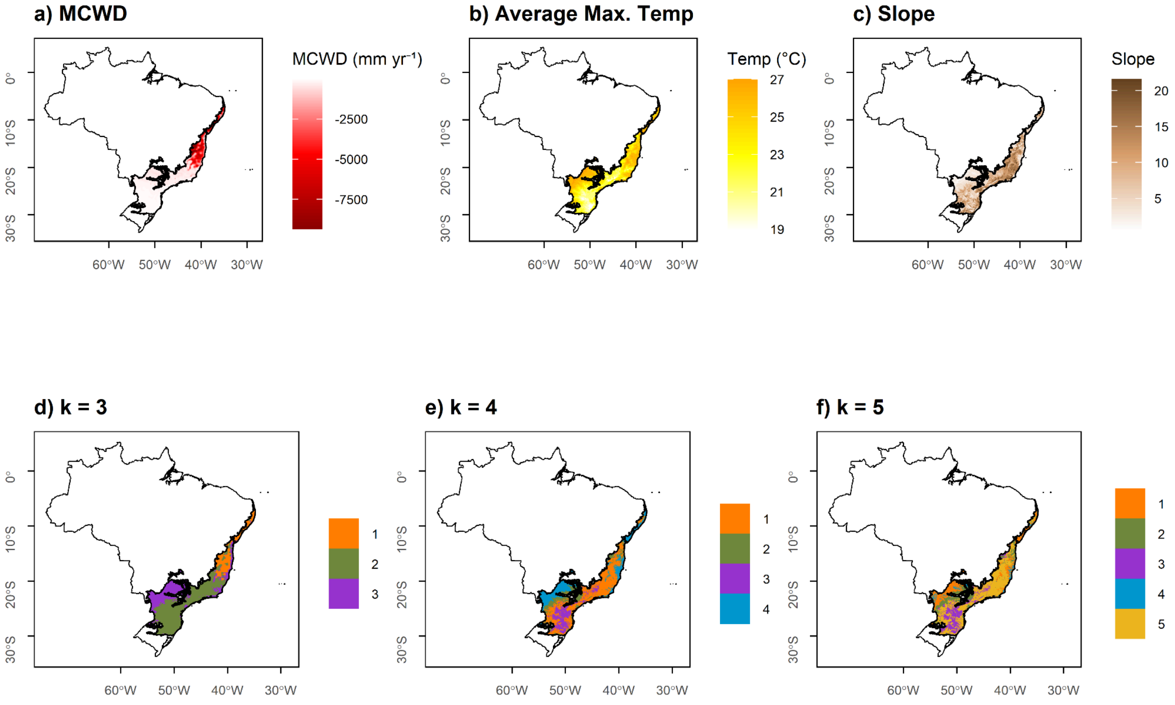

Supplementary Figure 9. Maps showing the spatial variation of the (a) average maximum cumulative water deficit (MCWD) between 1985-2020; (b) the average maximum temperature between 1985-2020; (c) terrain slope; and the k-means clustering test with (d) 3 regions, (e) 4 regions and (f) 5 regions.

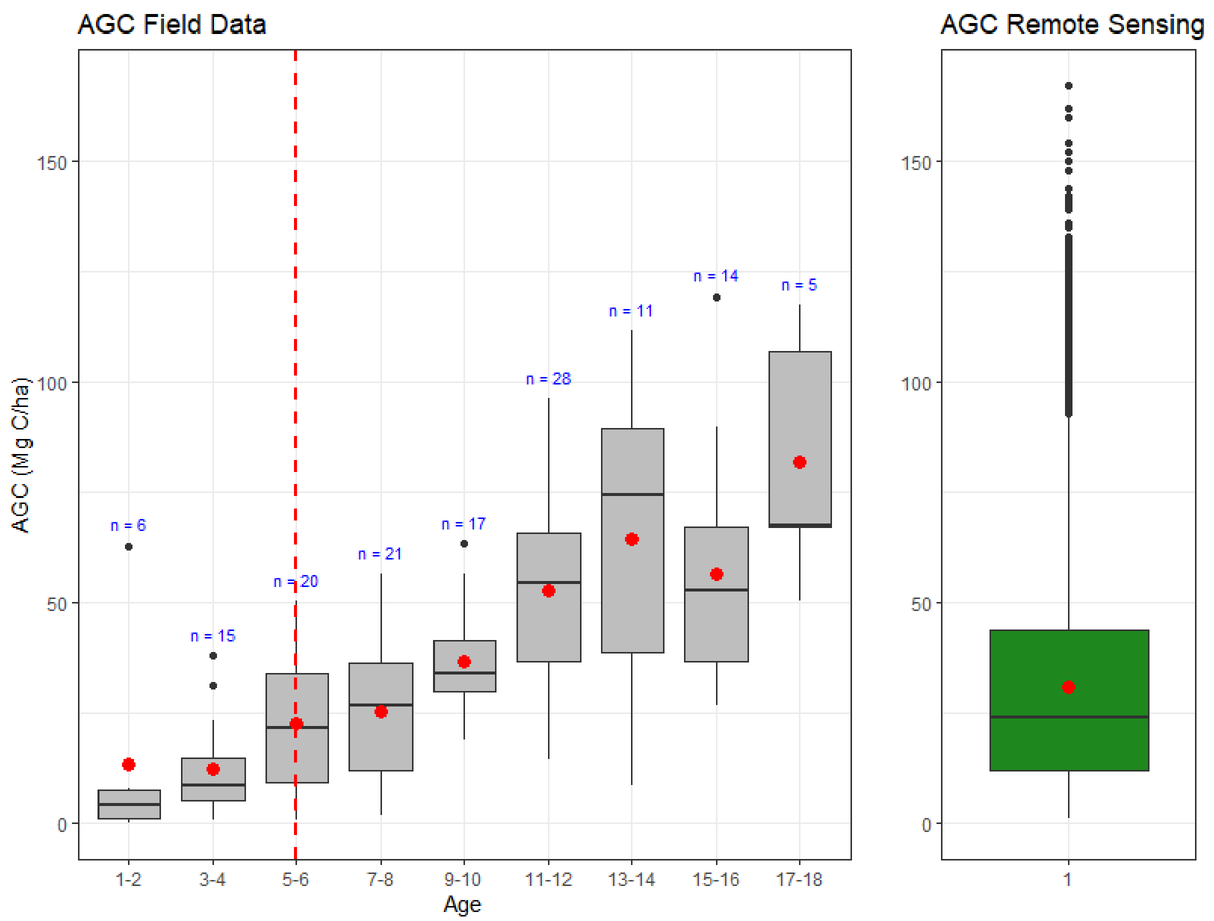

Supplementary Figure 10. Distribution of AGC (Mg C/ha) by secondary forest age based on field observations and for one-year-old secondary forest from remote sensing. The dashed red line shows where the AGC of field observation and remote sensing are not statistically significantly different.

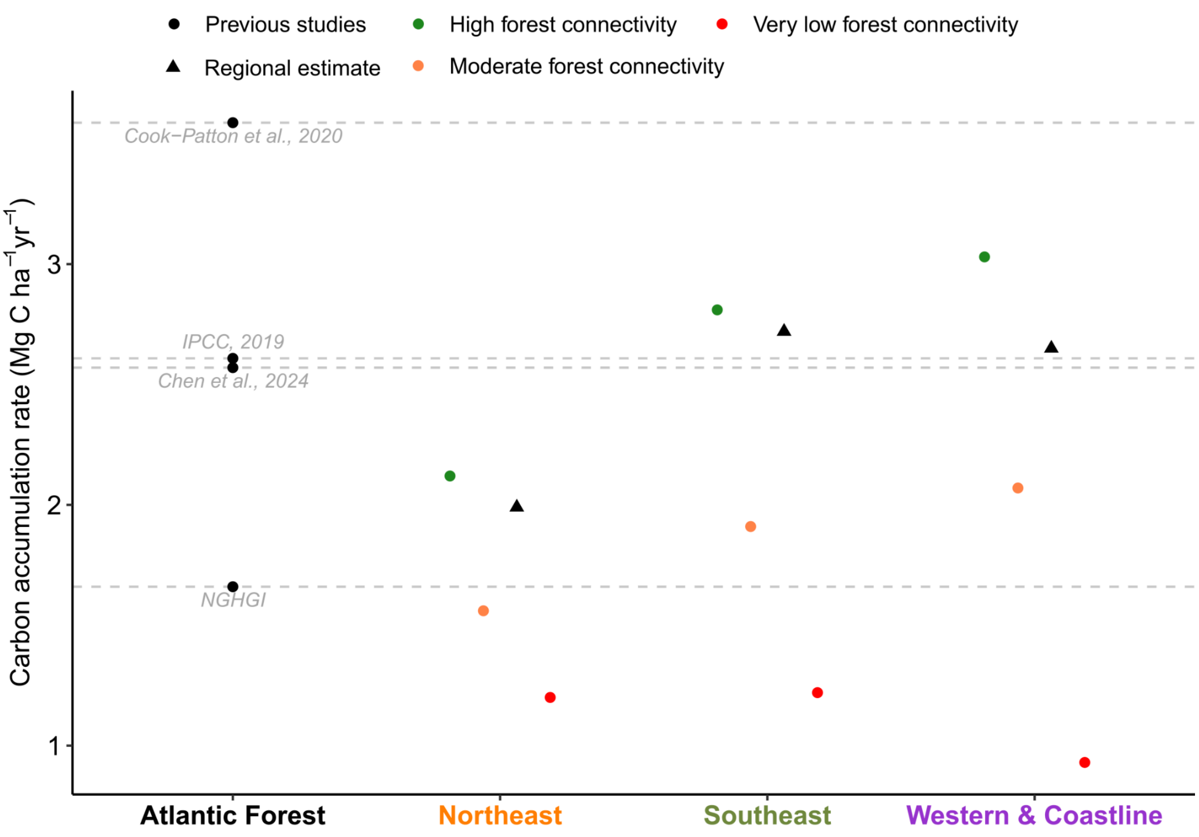

Supplementary Figure 11. Comparison of average regrowth rates (forest age <= 20 years) from this study and other literature estimates for the Atlantic Forest. The coloured points and black triangles are estimates from this study and show the average regrowth rate for each specific region and landscape fragmentation category. The black points and their respective grey dashed lines denote the average regrowth estimates from the whole Brazilian Atlantic Forest from Cook-Patton et al (2020), Chen et al, (2024) and IPCC, (2019) and NGHGI. To facilitate visualization in the figure, the uncertainty estimate can be found in the Supplementary Table 2.

Supplementary Table 1. Environmental characteristics for each Brazilian Atlantic Forest region

**Region**	**Average MCWD (SD)**	**Average Max. Temperature (SD)**	**Average Slope (SD)**
Northeast	-6288.4 (1967.43)	25.5 (0.91)	10.1 (6.44)
Southeast	-565.7 (1021.68)	22.7 (1.43)	12.8 (6.88)
Western & Coastline	-1079.9 (1059.51)	25.7 (0.73)	5.1 (3.96)

Supplementary Table 2. Average GLS model outputs for each Brazilian Atlantic Forest region

Region	Predictors	Standardized beta coefficients	CI low	CI upper	p-value
**Northeast**	Intercept	-0.02	-11.09	11.04	0.02
	Age	0.33	0.27	0.39	<0.001
	Size of fragment	-0.01	-0.07	0.05	0.52
	Forest connectivity	0.24	0.18	0.31	<0.001
	Av. Max. Temperature	0.00	-0.10	0.09	0.52
	MCWD	0.14	0.05	0.23	0.02
	Terrain slope	0.06	-0.01	0.12	0.22
**Southeast**	Intercept	-0.01	-0.11	0.08	<0.001
	Age	0.20	0.16	0.23	<0.001
	Size of fragment	0.03	0.00	0.07	0.16
	Forest connectivity	0.31	0.27	0.35	<0.001
	Av. Max. Temperature	0.02	-0.04	0.08	0.43
	MCWD	0.09	0.03	0.16	0.03

	Terrain slope	0.04	0.00	0.08	0.13
**Western and Coastline**	Intercept	-0.05	-0.26	0.16	<0.001
	Age	0.28	0.23	0.33	<0.001
	Size of fragment	0.03	-0.02	0.07	0.30
	Forest connectivity	0.28	0.23	0.34	<0.001
	Av. Max. Temperature	-0.03	-0.10	0.05	0.39
	MCWD	0.02	-0.06	0.11	0.45
	Terrain slope	0.02	-0.04	0.07	0.34

Supplementary Table 3. Average regrowth rates (Mg C ha-1 yr-1) in young secondary forests (<=20yrs) in the Atlantic Forest from this study and other sources

	Region	Average growth (<=20yr)	Uncertainty	Reference
	Atlantic Forest	3.59	0.12	Cook-Patton et al., 2020
	Atlantic Forest	2.57	-	Chen et al., 2024
	Atlantic Forest	2.61	-	IPCC, 2019
	Atlantic Forest	1.66	-	NGHGI
	Northeast	1.99	0.25	This study
	Southeast	2.72	0.67	This study
	Western & Coastline	2.65	0.67	This study
	By the landscape fragmentation category			
Very low forest connectivity	Northeast	1.20	0.37	This study
Moderate forest connectivity	Northeast	1.56	0.33	This study
High forest connectivity	Northeast	2.12	0.28	This study
Very low forest connectivity	Southeast	1.22	0.40	This study
Moderate forest connectivity	Southeast	1.91	0.32	This study
High forest connectivity	Southeast	2.81	0.95	This study
Very low forest connectivity	Western & Coastline	0.93	0.34	This study
Moderate forest connectivity	Western & Coastline	2.07	0.40	This study
High forest connectivity	Western & Coastline	3.03	0.81	This study

Supplementary Table 4. Estimated carbon stocks of standing secondary forests in 2020 under the two conservation scenarios for 2020, 2030 and 2050. Values are in Tg C (95% confidence interval)

Scenario	2020 (Tg C)	2030 (Tg C)	2050 (Tg C)
S1	385 (358,413)	517 (496, 539)	636 (617, 565)
S2	321 (307, 336)	379 (366, 393)	435 (423, 447)
