# Supplementary material for: Forest connectivity boosts carbon recovery in regenerating Atlantic Forests
Source: Commun Earth Environ. 2026 Apr 9;7(1):627. doi: 10.1038/s43247-026-03480-5 (PMC13427630; doi:10.1038/s43247-026-03480-5)
Supplement: Supplementary file 1 — Transparent Peer Review File [file 43247_2026_3480_MOESM1_ESM.pdf]

nature portfolio

Peer Review File

Forest connectivity boosts carbon recovery in regenerating Atlantic Forests

Corresponding Author: Dr Thais Rosan

This file contains all editorial decision letters in order by version, followed by all author rebuttals in order by version.

Version 0:

Decision Letter:

** Please ensure you delete the link to your author home page in this e-mail if you wish to forward it to your coauthors **

Dear Dr Rosan,

Please allow us to apologize for sending this late decision on your manuscript titled "Forest Connectivity Boosts Carbon Recovery in Regenerating Atlantic Forests". It has now been seen by 2 reviewers, and we include their comments at the end of this message. They find your work of interest, but some important points are raised. We are interested in the possibility of publishing your study in Communications Earth & Environment, but would like to consider your responses to these concerns and assess a revised manuscript before we make a final decision on publication.

We therefore invite you to revise and resubmit your manuscript, along with a point-by-point response that takes into account the points raised. Please highlight all changes in the manuscript text file.

Please submit your point-by-point responses as a separate file, distinct from your cover letter where you can add responses to the Editors' comments that you do not want to be made available to the reviewers. Word files are preferred. We recommend that any figures, tables or graphs that are included in the response to reviewers are also included in the main article or Supplementary Information.

We are committed to providing a fair and constructive peer-review process. Please don't hesitate to contact us if you wish to discuss the revision in more detail.

Please use the following link to submit your revised manuscript, point-by-point response to the referees' comments (which should be in a separate document to any cover letter), a tracked-changes version of the manuscript (as a PDF file) and the completed checklist:
Link Redacted
** This url links to your confidential home page and associated information about manuscripts you may have submitted or be reviewing for us. If you wish to forward this email to co-authors, please delete the link to your homepage first **

We hope to receive your revised paper within six weeks; please let us know if you aren't able to submit it within this time so that we can discuss how best to proceed. If we don't hear from you, and the revision process takes significantly longer, we may close your file. In this event, we will still be happy to reconsider your paper at a later date, as long as nothing similar has been accepted for publication at Communications Earth & Environment or published elsewhere in the meantime.

Please do not hesitate to contact us if you have any questions or would like to discuss these revisions further. We look forward to seeing the revised manuscript and thank you for the opportunity to review your work.

Best regards,

Rossella Guerrieri, PhD
Editorial Board Member
Communications Earth & Environment
orcid.org/0000-0001-5247-0432

Mengjie Wang
Associate Editor, Communications Earth & Environment
Consulting Editor, Communications Sustainability
Bluesky: @commsearth.nature.com; @ commssustain.nature.com

EDITORIAL POLICIES AND FORMATTING

We ask that you ensure your manuscript complies with our editorial policies. Please ensure that the following formatting requirements are met, and any checklist relevant to your research is completed and uploaded as a Related Manuscript file type with the revised article.

For Manuscripts that fall into the following fields:
• Behavioural and social science
• Ecological, evolutionary & environmental sciences
• Life sciences
An updated and completed version of our Reporting Summary must be uploaded with the revised manuscript
You can download the form here:
https://www.nature.com/documents/nr-reporting-summary.pdf

Furthermore, please align your manuscript with our format requirements, which are summarized on the following checklist: Communications Earth & Environment formatting checklist

and also in our style and formatting guide Communications Earth & Environment formatting guide .

*** DATA: Communications Earth & Environment endorses the principles of the Enabling FAIR data project (http://www.copdess.org/enabling-fair-data-project/). We ask authors to make the data that support their conclusions available in permanent, publically accessible data repositories. (Please contact the editor if you are unable to make your data available).

All Communications Earth & Environment manuscripts must include a section titled "Data Availability" at the end of the Methods section or main text (if no Methods). More information on this policy, is available at http://www.nature.com/authors/policies/data/data-availability-statements-data-citations.pdf.

In particular, the Data availability statement should include:
- Unique identifiers (such as DOIs and hyperlinks for datasets in public repositories)
- Accession codes where appropriate
- If applicable, a statement regarding data available with restrictions
- If a dataset has a Digital Object Identifier (DOI) as its unique identifier, we strongly encourage including this in the Reference list and citing the dataset in the Data Availability Statement.

DATA SOURCES: All new data associated with the paper should be placed in a persistent repository where they can be freely and enduringly accessed. We recommend submitting the data to discipline-specific, community-recognized repositories, where possible and a list of recommended repositories is provided at http://www.nature.com/sdata/policies/repositories.

If a community resource is unavailable, data can be submitted to generalist repositories such as figshare or Dryad Digital Repository. Please provide a unique identifier for the data (for example a DOI or a permanent URL) in the data availability statement, if possible. If the repository does not provide identifiers, we encourage authors to supply the search terms that will return the data. For data that have been obtained from publically available sources, please provide a URL and the specific data product name in the data availability statement. Data with a DOI should be further cited in the methods reference section.

Please refer to our data policies at http://www.nature.com/authors/policies/availability.html.

REVIEWER COMMENTS:

Reviewer #1 (Remarks to the Author):

General comments:
The manuscript by Rosan et al assesses how forest connectivity influences regrowth rates and aboveground carbon accumulation in secondary forests within the Brazilian Atlantic Forest biome. The study highlights the importance of landscape structure in shaping carbon dynamics and its relevance for forest restoration, climate mitigation and biodiversity conservation.

I found the topic compelling. The manuscript is well written and the ideas are coherent and well-articulated. The methods are solid and generally clearly explained. The results provide convincing evidence to support climate and forest policy and management. A structured and comprehensive discussion, acknowledging some of the analysis limitations and comparing with existing literature and reference quantifications, is provided.
I suggest some minor revisions to improve the manuscript:
• The study centres on forest connectivity. It will be helpful to visualise the patterns of the FAD metric in the 3 regions considered. I suggest adding a map showing this metric, ideally alongside the other variables considered (forest age, fragment size, MCWD, slope etc.).
• Additional justifications for the methodological choices are needed, such as 3 as the number of clusters to separate regions, the moving window size of 35 pixels, the choice of the FAD to reflect connectivity instead of other landscape / patch-level metrics, such as cohesion index or edge density.
• It is unclear at what spatial resolution and with what projection system were the maps processed.
• The environmental variables are derived from datasets of various spatial resolutions. Specifically, MCWD and temperature are from ERA5, whose monthly aggregate is at 27,830 m resolution. This is very coarse and lacks the fine-scale variability captured with the other datasets. How was this accounted for? Has there been any downscaling applied to match the finer resolution maps? If not, what would be the effects on the results?
• Pearson correlation is used for testing correlation among forest age and environmental variables. This test is parametric and requires a normal distribution, which I suspect would not be the case for some of the variables used. If so, I suggest checking with a non-parametric test, such as the rank-based Spearman, as the results could be substantially different from Pearson test.
• What formula was used to calculate MCWD?
• Information on the field data used for correcting and validating AGC are missing (data source, plot size, etc.).
• The pixel-based AGB estimates in the ESA CCI product are associated with large standard deviations as forests become dense. How were these pixel uncertainties accounted for across the range of forest ages?
• I understand the same environmental/climate conditions are maintained under the scenarios assessed (apart from forest age). Is that correct? If so, this needs to be clarified.
• The inset maps in Figure 1 and 2 are helpful, but lack contextualisation for readers not familiar with the location of the Brazilian Atlantic forests. I suggest adding a coastline or the country boundaries.

Detailed comments:
Below more detailed comments, with line numbering generated on my side (I guess it was an oversight, but the lines were not numbered in the original manuscript and supplementary material I downloaded):
• Line 57: For readers not familiar with the Brazilian context, perhaps it may be helpful to clarify that “biome” is in reference to Brazil’s national classification, not WWF/Olson's definition for intercontinental terrestrial regions under similar climate and vegetation.
• Line 77-78: I suggest rewording the sentence to only focus on the objectives and not present results at this stage.
• Line 200: There are 3 scenarios announced, but only 2 presented.
• Line 484: There may be situations, where the forest fragment may have a size of 1 ha but is elongated and narrow, and thus not exactly matching the 100m square pixels of ESA CC biomass maps.
• Line 502: A reference to IPCC citation need to be inserted.
• Line 238: Scenario 3 is presented but not appearing in Figure 4.
• Line 505: I understand “SF” means “secondary forest”, but this acronym was not presented before.
• Line 545: Past tense needed: “edit” should be replaced by “edited”
• Line 547: The sentence is difficult to follow (too many “to”): “ …to calculate the monthly water deficit to obtain the annual MCWD, to use the monthly spatial average (1985-2020) evapotranspiration from TerraClimate”.

Conclusion:
The manuscript assesses how forest recovery and carbon accumulation are affected along a forest fragmentation gradient, providing insights into the resilience of the Atlantic forests and highlighting the importance of landscape consideration in forest policy and management. I recommend this paper for publication after minor revisions.

Reviewer #2 (Remarks to the Author):

In this paper the authors combine a number of remote sensing datasets (land classification from MapBiomas, biomass from ESA) to model the recovery of aboveground carbon stocks through time relative to old-growth forests in the Brazilian Atlantic Forest. They find regional differences in recovery rates, and most importantly that landscape connectivity is a very important driver of recovery, with secondary forests in highly connected landscapes recovering far more swiftly than those in low-connectivity landscapes. The paper reads well, tackles an important question at a regional scale with useful implications for restoration planning, and is presented really nicely, good job!
I like the paper, and the way the findings are represented, but I have a couple of important points that need to be addressed.
- Medians when modelling recovery – I have looked through the methods of this paper, and the previous papers cited in this one that use the same methodology, and I cannot find any justification for using only the median value for each potential SF age when fitting the growth curve. You state that you have ~400,000 SF polygons, so why not put all the data into the growth model, instead of reducing all that data/variation down to just 1 summary value per potential age year? This confuses me. There are arguments you could make (e.g. remote sensing biomass estimates are inaccurate so this balances it out), but I don’t see any justification in the paper for only using medians. I think a clear and convincing argument needs to be

presented for why all the analysis relies on median values, and the raw data needs to be shown somewhere (at least in the supplementary).
- 3 regions from K means-clustering – I'll start by saying I am not an expert on the Brazilian Atlantic Forest, so I am happy to be proved wrong here. However, I did not read any justification for the K-means clustering approach to create different regions. Maybe you already tested a number of different potential K's (e.g. 2, 3, 4, 5) and found that k= 3 did the best job, if so great, but if not, I do not understand why there are 3 pre-determined regions. Perhaps these regional groupings are common/well known, if so you should include information about the regions and why they have been used. If not, you need to include more information on how you did the k-means clustering and how you selected k = 3. I did a quick search of recent papers on the Brazilian Atlantic Forest and found none referring to 3 regions similar to those presented in this paper. I'm not discrediting the three regions, just find that currently there is not an adequate explanation as to how they were chosen, so if the regions are to be kept a proper explanation will need to be added. I also think a value and growth curve for the entire Atlantic Forest would be a useful addition too.
Some additional specific comments:

• L27 – can you give this as a % increase or something relative to make it more tangible to the reader?
• L64 - *forest
• 92-97 – pretty lengthy opening sentence, suggest splitting into two
• 113-117 – this is phrased slightly confusingly, 'to leave the secondary forests standing in 2020' – do you mean that it will take 47 years from 2020 for secondary forests to reach 95% of old-growth AGC, or just that it takes 47 years in total for a secondary forest to regrow to 95% of old-growth AGC – if the later, I suggest phrasing it more clearly
• 159 – why group them into categories – why not just model the continuous effect of connectivity on regrowth?
• 216 – useful to put a % increase to contextualise it – e.g. increase 35% to 517
• 238 – this is the first we are hearing of Scenario 3 – I am assuming it was a previous version that was removed
• Table 1 – if you are sticking with the 2 scenarios, this table is not necessary, since you report all the values within the text. I suggest removing it or moving to supplementary
• 364 – To me this does not flow right by introducing new results and figures in the middle of your discussion. I think these sections need to be moved up in the main paper
• 366-368 – and I think I am right in saying that all your presented results are for AGB only? So SFs would have even less carbon compared to old-growth if accounting for below-ground carbo also
• 407 – again, it's a nice figure, but not one that should be introduced in the discussion. I suggest this one gets moved to supplementary
• 437 – you could also allude to new datasets that will be soon be released that should help with this (e.g. BIOMASS)
• 551 – I'm not hugely familiar with the Brazilian Atlantic Forest, but interested in why you split the biome into three regions, what's the rationale for this? Is it generally accepted that there are 3 distinct regions? Did you test other numbers on the clustering?
• 572- how come you grouped the old growth and secondary forests into one mask for the fragmentation analysis, wont this put secondary and old-growth forest on the same level in terms of connectivity value? It's likely that the proportion of surrounding forest that is old growth vs secondary will have a strong impact on biomass recovery (e.g. an area that is surrounded by 70% forest, all of which is old-growth, is likely to be getting more dispersal etc. than an area surrounded by 80% forest but most of this is secondary?)
• 577 – what is the dominant value? Median? Better to state this explicitly
• 588 – equation format has not come through correctly in the PDF

** Visit Nature Portfolio's author and referees' website at www.nature.com/authors for information about policies, services and author benefits**

Communications Earth & Environment is committed to improving transparency in authorship. As part of our efforts in this direction, we are now requesting that all authors identified as 'corresponding author' create and link their Open Researcher and Contributor Identifier (ORCID) with their account on the Manuscript Tracking System prior to acceptance. ORCID helps the scientific community achieve unambiguous attribution of all scholarly contributions. You can create and link your ORCID from the home page of the Manuscript Tracking System by clicking on 'Modify my Springer Nature account' and following the instructions in the link below. Please also inform all co-authors that they can add their ORCIDs to their accounts and that they must do so prior to acceptance.
https://www.springernature.com/gp/researchers/orcid/orcid-for-nature-research

For more information please visit http://www.springernature.com/orcid

If you experience problems in linking your ORCID, please contact the Platform Support Helpdesk.

Version 1:

Decision Letter:

** Please ensure you delete the link to your author home page in this e-mail if you wish to forward it to your coauthors **

Dear Dr Rosan,

Your manuscript titled "Forest Connectivity Boosts Carbon Recovery in Regenerating Atlantic Forests" has now been seen by our reviewers, whose comments appear below. In light of their advice we are delighted to say that we are happy, in principle, to publish a suitably revised version in Communications Earth & Environment.

We therefore invite you to revise your paper one last time to address the remaining concerns of our reviewers. At the same time we ask that you edit your manuscript to comply with our format requirements and to maximise the accessibility and therefore the impact of your work.

EDITORIAL REQUESTS:

Please review our specific editorial comments and requests regarding your manuscript in the attached "Editorial Requests Table".

*****Please take care to match our formatting and policy requirements. We will check revised manuscript and return manuscripts that do not comply. Such requests will lead to delays. *****

Please outline your response to each request in the right hand column. Please upload the completed table with your manuscript files as a Related Manuscript file.

If you have any questions or concerns about any of our requests, please do not hesitate to contact me.

SUBMISSION INFORMATION:

In order to accept your paper, we require the files listed at the end of the Editorial Requests Table; the list of required files is also available at https://www.nature.com/documents/commsj-file-checklist.pdf .

OPEN ACCESS:

Communications Earth & Environment is a fully open access journal. Articles are made freely accessible on publication. For further information about article processing charges, open access funding, and advice and support from Nature Portfolio, please visit https://www.nature.com/commsenv/open-access

At acceptance, you will be provided with instructions for completing the open access licence agreement on behalf of all authors. This grants us the necessary permissions to publish your paper. Additionally, you will be asked to declare that all required third party permissions have been obtained, and to provide billing information in order to pay the article-processing charge (APC).

Please use the following link to submit the above items:
Link Redacted
** This url links to your confidential home page and associated information about manuscripts you may have submitted or be reviewing for us. If you wish to forward this email to co-authors, please delete the link to your homepage first **

We hope to hear from you within two weeks; please let us know if you need more time.

Best regards,

Rossella Guerrieri, PhD
Editorial Board Member
Communications Earth & Environment
orcid.org/0000-0001-5247-0432

Mengjie Wang
Associate Editor, Communications Earth & Environment
Consulting Editor, Communications Sustainability
Bluesky: @commsearth.nature.com; @commssustain.nature.com

REVIEWERS' COMMENTS:

Reviewer #1 (Remarks to the Author):

I thank the authors for the careful clarification.

Regarding clustering, I was curious whether the authors used specific techniques to identify natural grouping in the data (e.g. Elbow method, silhouette method etc.). These would provide more robust justification to determine an optimal number of clusters, as they aim to maximise cohesion within groups and separation among them. But I think the rationale for taking 3 clusters has been well explained, as I understand the intention is to highlight known bioclimatic regions.

Overall, I am satisfied with the Authors' response and revisions. All the concerns I raised were thoughtfully addressed and the clarity of the manuscript has been substantially improved. I recommend this paper for publication.

Reviewer #2 (Remarks to the Author):

The authors have done a good job responding to all my comments, making some necessary changes and improvements, and explaining their analytical decisions. I am happy that the manuscript is now ready for publication.
Below are just a small number of changes I noticed were required when re-reading the manuscript in full:
L373 – stopping not 'stop'
L383-385 – you should also add in that it takes far longer to achieve old-growth biodiversity (if at all) than it does carbon stocks
L407 – y? typo here I think
Fig 1 – thank you for including the biome-wide growth curve as I suggested. Looking at the figure, I understand if the authors would rather remove this black curve from the main text figure to keep continuity with the three models presented in other figures. If so, please put the biome-wide curve in the supplementary, and revert to just the three regions in Fig 1 (I do not mind which – the authors can choose what option they prefer)

** Visit Nature Portfolio's author and referees' website at www.nature.com/authors for information about policies, services and author benefits**

Open Access This Peer Review File is licensed under a Creative Commons Attribution 4.0 International License, which permits use, sharing, adaptation, distribution and reproduction in any medium or format, as long as you give appropriate credit to the original author(s) and the source, provide a link to the Creative Commons license, and indicate if changes were made.
In cases where reviewers are anonymous, credit should be given to 'Anonymous Referee' and the source.
The images or other third party material in this Peer Review File are included in the article's Creative Commons license, unless indicated otherwise in a credit line to the material. If material is not included in the article's Creative Commons

license and your intended use is not permitted by statutory regulation or exceeds the permitted use, you will need to obtain permission directly from the copyright holder.
To view a copy of this license, visit https://creativecommons.org/licenses/by/4.0/

RESPONSE TO THE REVIEWER COMMENTS:

Colour code: The response to the reviewers is written in blue and the sentences added in the manuscript are in green. Line number refers to the tracked changed document.

Reviewer #1 (Remarks to the Author):

General comments:
The manuscript by Rosan et al assesses how forest connectivity influences regrowth rates and aboveground carbon accumulation in secondary forests within the Brazilian Atlantic Forest biome. The study highlights the importance of landscape structure in shaping carbon dynamics and its relevance for forest restoration, climate mitigation and biodiversity conservation.
I found the topic compelling. The manuscript is well written and the ideas are coherent and well-articulated. The methods are solid and generally clearly explained. The results provide convincing evidence to support climate and forest policy and management. A structured and comprehensive discussion, acknowledging some of the analysis limitations and comparing with existing literature and reference quantifications, is provided.

We thank you for the careful evaluation of our manuscript and for the positive and encouraging comments. We greatly appreciate the recognition of the study's relevance, methodological rigor, and clarity of presentation. Below, we address the reviewer's comments in detail.

I suggest some minor revisions to improve the manuscript:
• The study centres on forest connectivity. It will be helpful to visualise the patterns of the FAD metric in the 3 regions considered. I suggest adding a map showing this metric, ideally alongside the other variables considered (forest age, fragment size, MCWD, slope etc.).

Thank you for your suggestion. We agree that visualizing the spatial patterns of the FAD metric would be valuable. However, due to the elongated configuration of the Atlantic Forest biome and its highly fragmented landscape, characterized by predominantly small forest patches, visualizing the FAD metric and the secondary forest age at the 30m spatial resolution results in highly scattered pixels that are difficult to interpret visually. To attend the reviewer request and improve the visualization, we aggregated the pixels by a factor of 50 (~1.5 km2 x ~1.5 km2) spatial resolution using an average function to plot the whole Atlantic Forest with RStudio. In addition, we included small insets maps for each region at the native original spatial resolution of 30m to illustrate how scattered are the fragments within the landscape. These maps were added in the supplementary file as well pasted below.
The sample distribution for each secondary forest age and FAD category is presented in Supplementary Figures 1 and 3. The remaining environmental variables (MCWD, slope, and average maximum temperature) are shown in Supplementary Figure 9 in conjunction with the k-means clustering results. Because secondary forest fragments are very small, with an average size of 1.9 ha (SD 3.7ha), we present fragment size variation as a histogram in Supplementary Figure 5. For the reviewer's convenience, all of these new figures are also reproduced below.

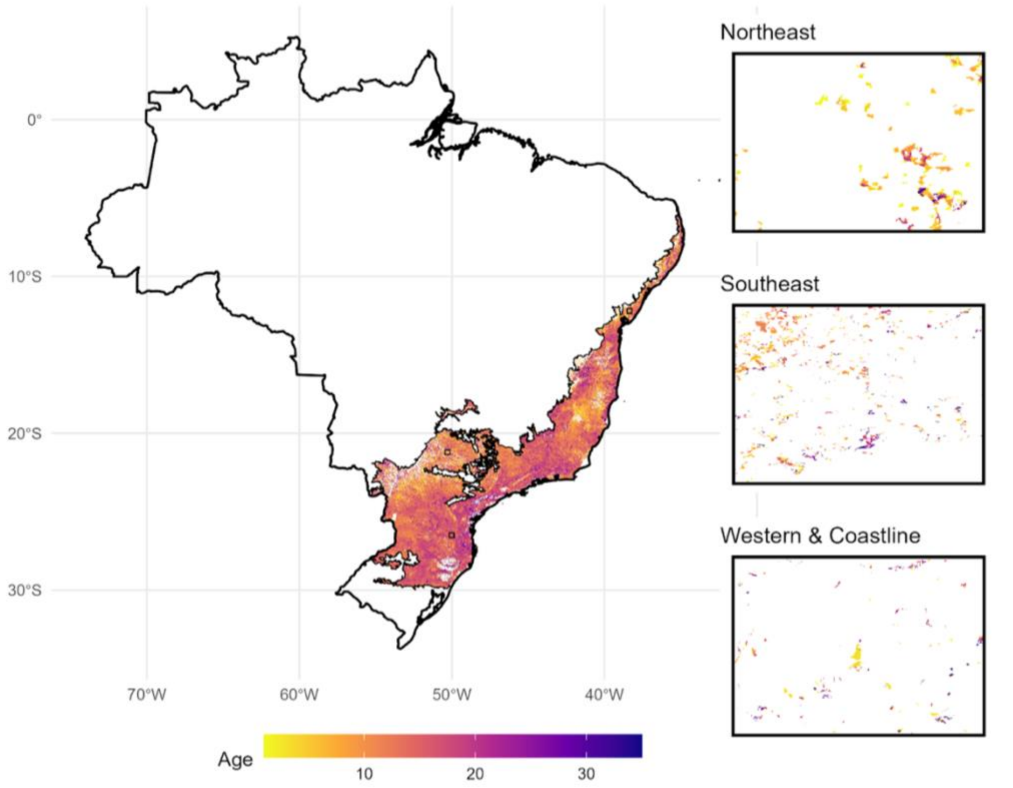

Supplementary Figure 2. Map showing the secondary forest age in the Atlantic Forest aggregated to a 1.5km^2 spatial resolution with an average age function. The inset maps show the original spatial resolution (30m) of the secondary forest age fragments for each region of Atlantic Forest defined in this study.

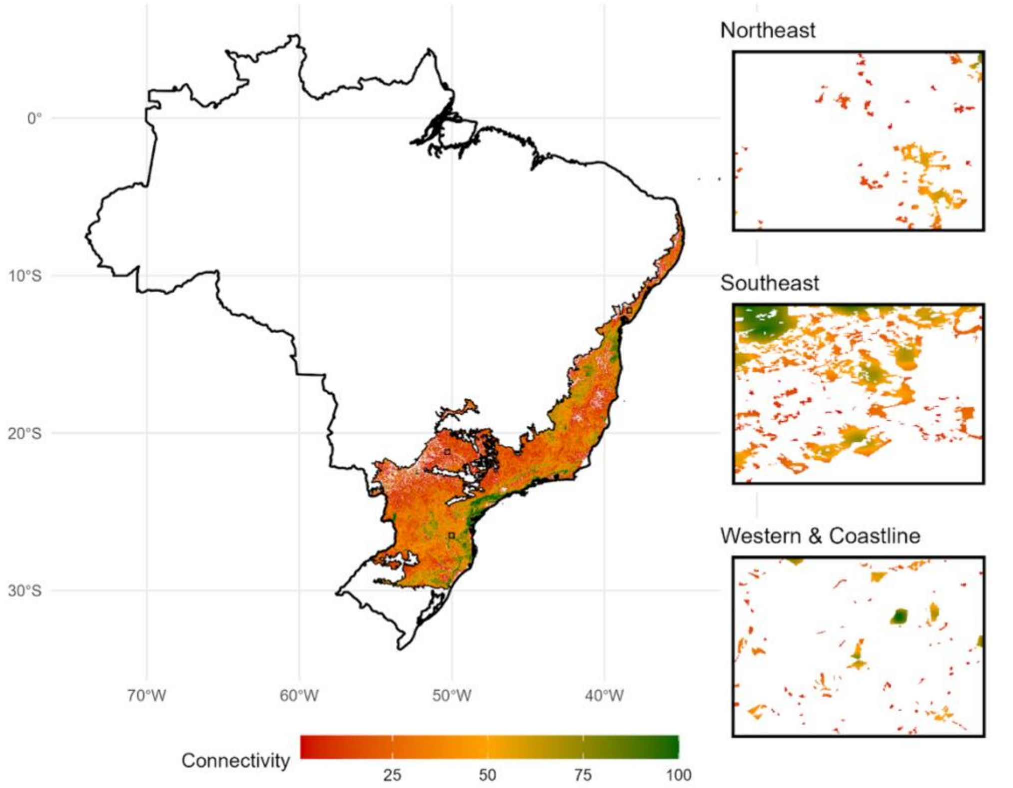

Supplementary Figure 4. Map showing the FAD connectivity metric in the Atlantic Forest aggregated to a 1.5km^2 spatial resolution with an average function. The inset maps show the

original spatial resolution (30m) of FAD for each region of Atlantic Forest defined in this study.

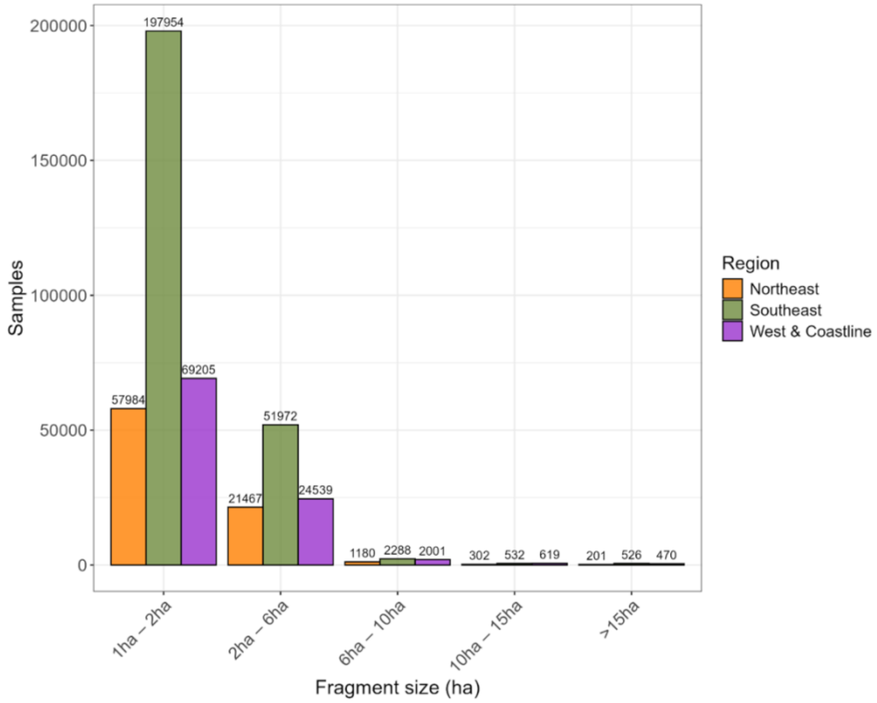

Supplementary Figure 5. Histogram with the sample distribution of the size of secondary forest fragments (ha) in each environmental region of the Atlantic Forest.

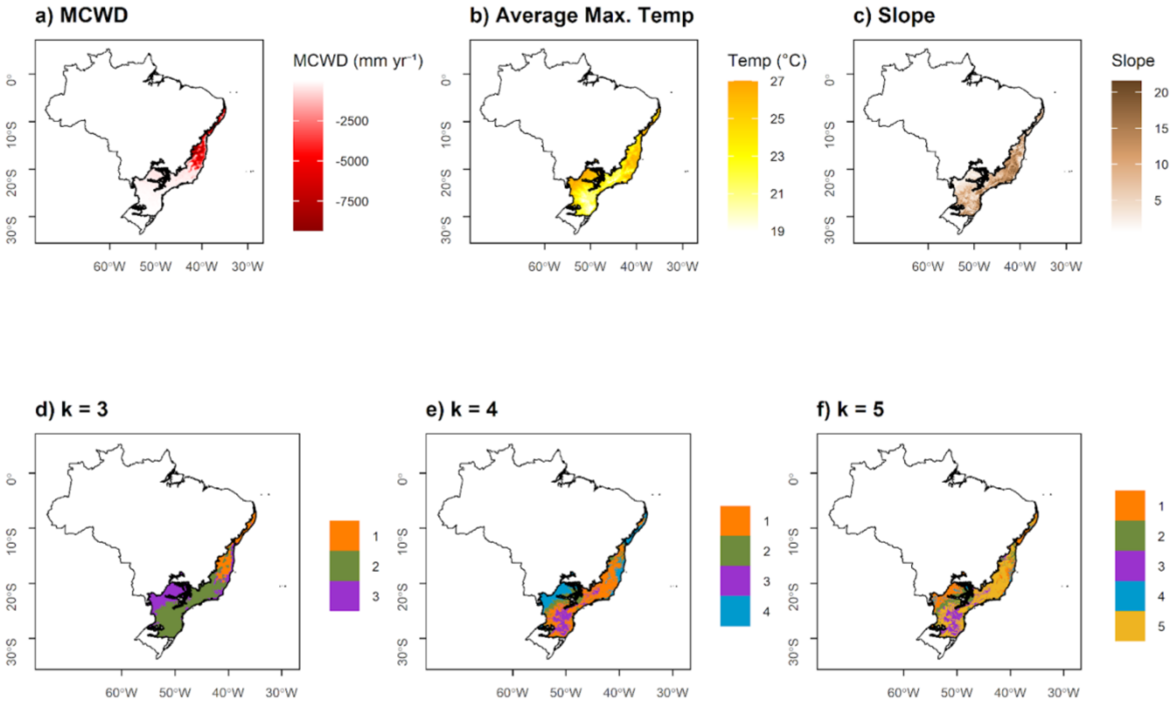

Supplementary Figure 9. Maps showing the spatial variation of the (a) average maximum cumulative water deficit (MCWD) between 1985-2020; (b) the average maximum temperature between 1985-2020; (c) terrain slope; and the k-means clustering test with (d) 3 regions, (e) 4 regions and (f) 5 regions.

• Additional justifications for the methodological choices are needed, such as 3 as the number of clusters to separate regions, the moving window size of 35 pixels, the choice of the FAD to reflect connectivity instead of other landscape / patch-level metrics, such as cohesion index or edge density.

Kmeans clustering

We thank the reviewer for this important comment and agree that additional justification for the choice of three clusters was needed. We have now expanded the Methods section to clarify the rationale for the regionalisation approach.

We used k-means clustering to identify environmentally coherent subregions within the Brazilian Atlantic Forest based on climatic and topographic variables known to influence forest structure, species composition, and successional dynamics. The Atlantic Forest spans a strong latitudinal and environmental gradient, ranging from cooler and wetter conditions in southern and high-elevation areas, to hotter and flatter conditions in western and coastal lowlands, and hotter and drier conditions in the northeast.

The choice of three clusters reflects this broad environmental heterogeneity and captures the dominant bioclimatic structure of the biome. Supplementary Table 1 summarizes the climatic and topographic characteristics of each region defined by the k-means clustering. In addition, we added supplementary figure 9 showing the spatial distribution of the environmental variables used in the clustering, as well as clustering results for k = 3, k = 4, and k = 5. Visual inspection of these results indicates that k = 3 provides the most parsimonious and ecologically interpretable regionalisation, whereas higher values of k primarily subdivide regions without introducing clearly distinct climatic domains.

Although there is no standardized k-means regionalisation for the Atlantic Forest, previous studies have identified three major functional and bioclimatic regions within the biome that broadly align with our clustering results[1,2], supporting the ecological relevance of this choice. We therefore consider k = 3 to be an appropriate balance between ecological realism, interpretability, and analytical tractability.

We edited the methods section to include the justification: “To determine the input variables for clustering the pixels based on the relationship with the AGC, we first calculated a correlogram to analyse collinear relationships between the environmental variables using a rank-based Spearman correlogram (Supplementary Figure 8). Variables with correlations exceeding ±0.5 were excluded to minimize multicollinearity. The final set of predictors for the k-means clustering included mean maximum temperature, mean maximum cumulative water deficit (MCWD), and terrain slope. The number of clusters was set to three to capture the dominant bioclimatic gradients of the Atlantic Forest. This choice reflects the broad environmental heterogeneity of the biome, which spans cooler and wetter southern and high elevation areas, hotter and flatter western and coastal lowlands, and hotter and drier conditions in the northeast. Clustering solutions with higher number of clusters (k=4 and k=5) primarily subdivided these broad regions without revealing clearly distinct climatic domains (Supplementary Figure 9). The resulting three regions were consistent with major functional and bioclimatic divisions of the Atlantic Forest reported in previous studies[56,57]. (Lines 639-656)

1. Silva, J. L. A., Souza, A. F. & Vitória, A. P. Mapping functional tree regions of the Atlantic Forest: how much is left and opportunities for conservation. *Environmental Conservation* **49**, 164–171 (2022).
2. Brown, J. L. *et al.* Seeing the forest through many trees: Multi-taxon patterns of phylogenetic diversity in the Atlantic Forest hotspot. *Diversity and Distributions* **26**, 1160–1176 (2020).

FAD metric

We chose a moving window size of 35 × 35 pixels to capture landscape-scale forest structure while balancing spatial resolution and computational feasibility. At 30 m pixel resolution, this corresponds to an area of approximately 1 km², which allows the analysis to reflect local landscape connectivity patterns without overgeneralizing across larger landscapes. Forest Area Density (FAD) was selected as an indicator of connectivity because it has been officially adopted by the Food and Agriculture Organization (FAO) for assessing forest structure and fragmentation at the landscape scale. Unlike patch-level metrics such as the cohesion index or edge density, FAD integrates information about the proportion of forested area within a defined neighbourhood and is particularly suited to analyses focused on connectivity across the landscape mosaics, rather than individual patches.

We edited the methods section, sentences in green shade were added in the manuscript text: "To assess landscape fragmentation, we used the Forest Area Density (FAD) index[1], which quantifies the proportion of forest cover within a given neighbourhood and is the recommended Food and Agriculture Organization (FAO) indicator for assessing forest connectivity at landscape level." (Lines 660-663)

"We used a moving window of 35 × 35 pixels (~ 1km²) to calculate the FAD values. This window size was chosen to capture landscape-scale patterns of connectivity structure in an already highly fragmented landscape such as the Atlantic Forest." (Lines 672-675)

• It is unclear at what spatial resolution and with what projection system were the maps processed.

All maps were processed at their native spatial resolution, except for the climate layers, which were resampled to a 100 m spatial resolution using the nearest-neighbour method. We have included a clarifying sentence in the text:

"It is important to note that the ERA-5 derived climate layers (e.g., temperature, MCWD) and other environmental variables such as elevation and slope were resampled in Google Earth Engine to a 100 m grid using the default nearest-neighbour resampling method to overlay the secondary forest fragments layer for zonal statistics calculation. As such, the resulting fine-resolution raster retains the original coarse-scale values of the climate data and does not reconstruct sub-pixel variability. This limitation means that fine-scale climatic heterogeneity within the original coarse pixel footprint may not be captured." (Lines 627-635)

The secondary forest map was processed at a 30 m spatial resolution and converted to vector format, as stated in the Methods section:
"The remaining pixels of secondary forest age were grouped by age and location using the 'RegionGroup' function from ArcPy and converted to a vector format. Then, only the secondary forest fragments that were greater than 1 ha were selected for further analysis to match the spatial resolution of the aboveground biomass product (100m)." (Lines 521-535)
These vector files were subsequently used to extract all zonal statistics for each fragment. For additional clarity, we added the following sentence in the Methods section:
"This vector file of secondary forest fragments, using the WGS 84 projection system, was used to extract all zonal statistics required for the analyses described in the sections below." (Lines 536-538)

• The environmental variables are derived from datasets of various spatial resolutions. Specifically, MCWD and temperature are from ERA5, whose monthly aggregate is at 27,830 m resolution. This is very coarse and lacks the fine-scale variability captured with the other datasets. How was this accounted for? Has there been any downscaling applied to match the finer resolution maps? If not, what would be the effects on the results?

Thank you for raising this important point. We used ERA5-derived variables (e.g. MCWD, temperature) within Google Earth Engine (GEE) and resampled these layers to a 100 m grid to match our other spatial datasets. By default, when GEE reprojects or resamples image data to a new grid, it applies a nearest-neighbour method for continuous rasters, which preserves the original pixel values. Thus, rather than applying a statistical downscaling or interpolation, our procedure effectively assigned the original coarse-resolution ERA5 value to each 100 m pixel. We acknowledge that this approach does not introduce new fine-scale spatial detail and cannot reconstruct sub-grid variability that was not present in the original data. We added the following sentence in the methods section "It is important to note that the ERA-5 derived climate layers (e.g., temperature, MCWD) and other environmental variables such as elevation and slope were resampled in Google Earth Engine to a 100m grid using the default nearest-neighbour resampling method to overlay the secondary forest fragments layer for zonal statistics calculation. As such, the resulting fine-resolution raster retains the original coarse-scale values of climate data and does not reconstruct sub-pixel variability. This limitation means that fine-scale climatic heterogeneity within the original coarse pixel footprint may not be captured." (Lines 627-635)

• Pearson correlation is used for testing correlation among forest age and environmental variables. This test is parametric and requires a normal distribution, which I suspect would not be the case for some of the variables used. If so, I suggest checking with a non-parametric test, such as the rank-based Spearman, as the results could be substantially different from Pearson test.

Thank you for this comment. We agree that some variables may not meet the assumptions of the Pearson correlation. Accordingly, we re-analysed the correlations using the rank-based Spearman method. While some correlation values differ slightly, the overall selection of environmental variables remains unchanged. We have updated the manuscript text and supplementary figure to reflect the Spearman correlations.

The edited section of the methods now states as: “To determine the input variables for clustering the pixels based on the relationship with the AGC, we first calculated a correlogram to analyse collinear relationships between the environmental variables using a rank-based Spearman correlogram (Supplementary Figure 8). Variables with correlations exceeding ±0.5 were excluded to minimize multicollinearity.” (Lines 639-644)

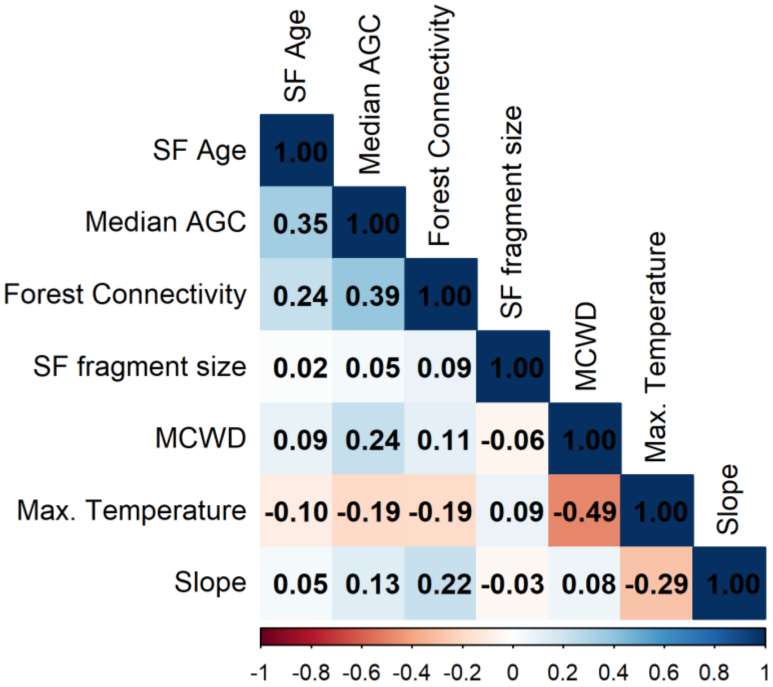

Supplementary Figure 8. Correlogram showing ranked-based Spearman R value between the environmental variables and secondary forest age. The values within each box show Spearman R value, and the colour shows the relative degree and sign of the relationship between the variables. Variables with no shading were non-significant at the 95% confidence interval.

• What formula was used to calculate MCWD?

We calculated the Maximum Cumulative Water Deficit (MCWD) using monthly ERA5 precipitation data, following established approaches for tropical forests. Monthly water balance was computed using spatially explicit monthly evapotranspiration from TerraClimate (1985–2020), rather than assuming a constant evapotranspiration value, to better represent climatic heterogeneity across the Atlantic Forest. We added the equations in the methods section:

“The Maximum Cumulative Water Deficit (MCWD) is defined as the most negative value of climatological water deficit (CWD) over a year and was calculated using a script in R language based on the calculation of previous studies[48–50]. MCWD was calculated based on spatially explicit monthly ERA5 precipitation (P) data and spatially explicit monthly evapotranspiration (ET) from TerraClimate[55] (1985–2020). Rather than assuming a constant evapotranspiration value, we used the spatially explicit evapotranspiration to better

represent climatic heterogeneity across the Atlantic Forest. The CWD and MCWD was calculated as (equation 1); where *n* is month:

$$CWDn = CWDn - 1 + Pn - ETn;\ Max(CWDn) = 0;$$
$$CWD_0 = CWD_{12};\ MCWD = Min(CWD_1 \ldots CWD_{12}) \quad (1)$$

We then calculated the average MCWD for the whole period (1985-2020) for use in subsequent analysis." (Lines 609-626)

• Information on the field data used for correcting and validating AGC are missing (data source, plot size, etc.).

Thank you for your comment. We added the information in the methods section: "To support and validate the remote sensing-based estimates, we compiled a field database of aboveground carbon (AGC) derived from forest inventory data collected from 137 plots distributed across the Atlantic Forest biome between 2019 and 2024 as part of the project "Understanding restore forests for benefiting people and nature (NewFor)". Each plot covered an area of 30x30m, where all trees with diameter at breast height (DBH) greater than 5cm were measured. For each individual, DBH and total height were recorded, and species were identified to the species level when possible, or otherwise to the genus level, to allow assignment of woody density values from the Global Wood Density Database[49]. Aboveground biomass was estimated using allometric equations and converted to AGC assuming a carbon fraction of 0.47." (Lines 579-588)

54. Zanne, Amy E.; Lopez-Gonzalez, G.; Coomes, David A. et al. (2009). Data from: Towards a worldwide wood economics spectrum [Dataset]. Dryad. https://doi.org/10.5061/dryad.234

• The pixel-based AGB estimates in the ESA CCI product are associated with large standard deviations as forests become dense. How were these pixel uncertainties accounted for across the range of forest ages?

We agree with the reviewer that pixel-based AGB estimates from the ESA CCI product are associated with large standard deviations, particularly in dense forests. To mitigate these uncertainties and potential biases, we used median values in our analysis for the regrowth curves. The use of medians reduces the influence of extreme or biased values in both early and late successional stages, producing more stable and representative age-class estimates. We also addressed this in a later response to the use of medians in answer to reviewer 2 comment.

We acknowledge these uncertainties in the limitations section:

"A known limitation of remote sensing-derived biomass estimates is biomass saturation in high biomass forests and overestimation in low biomass regions44, which can be correlated with younger secondary forests. This overestimation may be attributed to the lower sensitivity of remote sensing-derived biomass models, which distinguish fine-scale variations in regrowth stages, leading to inflated biomass estimates for recently regenerating forests and result in lower growth rates" (Lines 455-461)

We further added that "Future studies should explore the integration of higher-resolution remote sensing datasets and active remote sensing techniques, such as LiDAR and new missions (e.g. BIOMASS, NISAR). For instance, BIOMASS is a P-Band sensor and therefore might help to reduce potential saturation effect in high-biomass tropical regions45,

however old-growth baselines are needed to help build these recovery curves. Other missions such as NASA-ISRO Synthetic Aperture Radar (NISAR) might also prove helpful to further improving aboveground biomass estimates as they have higher resolution. Therefore, refining land cover classification algorithms, improving biomass estimates derived from earth observation data, and incorporating field validation efforts will be essential for enabling that such data to be used effectively by end-users such by restoration monitoring communities[47]." (Lines 480-493)

• I understand the same environmental/climate conditions are maintained under the scenarios assessed (apart from forest age). Is that correct? If so, this needs to be clarified.

Yes, that is correct. All scenarios assume that environmental and climatic conditions remain constant over time, with forest age being the only variable that changes. We have clarified this assumption in the text.
Revised sentence in the manuscript
"Based on the relevance of secondary forests for climate mitigation and given the differences in the regrowth rates across the three regions, we modelled the aboveground carbon stocks of standing secondary forests in the Atlantic Forest biome in 2020. We then projected these stocks for 2030 and 2050 using the three regional regrowth models (Fig. 1) under two distinct scenarios, assuming constant environmental and climatic conditions and allowing only forest age to vary over time." (Lines 203-209)

• The inset maps in Figure 1 and 2 are helpful, but lack contextualisation for readers not familiar with the location of the Brazilian Atlantic forests. I suggest adding a coastline or the country boundaries.

Thank you, we updated Figure 1 and 3, adding the country boundaries for clarity.

Detailed comments:
Below more detailed comments, with line numbering generated on my side (I guess it was an oversight, but the lines were not numbered in the original manuscript and supplementary material I downloaded):

• Line 57: For readers not familiar with the Brazilian context, perhaps it may be helpful to clarify that "biome" is in reference to Brazil's national classification, not WWF/Olson's definition for intercontinental terrestrial regions under similar climate and vegetation.

Thank you, we added "as defined under Brazil's national biome classification" in the sentence. (Line 59)

• Line 77-78: I suggest rewording the sentence to only focus on the objectives and not present results at this stage.

Thank you for the suggestion. We changed the sentence from "In this study, we explicitly assess how forest connectivity influences regrowth curves, revealing the extent to which connected landscapes accumulate carbon more rapidly than fragmented ones" to "In this

study, we explicitly assess how forest connectivity influences regrowth curves, intending to understand its role in carbon accumulation across landscapes” (Lines 79-82)

• Line 200: There are 3 scenarios announced, but only 2 presented.

Thank you, it was an error. We remove the 3rd scenario in a previous edition and forgot to change in the text, now states just ‘under two distinct scenarios’.

• Line 484: There may be situations, where the forest fragment may have a size of 1 ha but is elongated and narrow, and thus not exactly matching the 100m square pixels of ESA CC biomass maps.

Thank you for your comment. The forest fragments were identified using MapBiomas 30m data, and only clusters of >= 12 pixels (~1ha) were selected on our analysis for the regrowth rates. This approach ensures that fragments are roughly comparable in size to a 100m ESA CCI Biomass pixel. We acknowledged that highly elongated or irregular fragments may not perfectly align with the pixel grid, however, because we used the median aboveground carbon (AGC) in further analysis from thousands of clusters >1ha, this aggregation helps mitigate potential biases from fragment shape and pixel misalignment.

We added the following sentence in the limitations section "Additionally, while we selected fragments ≥1 ha to roughly match the 100 m ESA CCI biomass pixels, some elongated or irregularly shaped fragments may not perfectly align with the pixel grid. However, using the median aboveground carbon from thousands of fragments helps mitigate potential biases due to fragment shape or pixel misalignment."

• Line 502: A reference to IPCC citation need to be inserted.
Thank you, we added it.

• Line 238: Scenario 3 is presented but not appearing in Figure 4.

Thank you, we removed the reference to Scenario 3.

• Line 505: I understand “SF” means “secondary forest”, but this acronym was not presented before.

Thank you, we removed the acronym to the full word ‘secondary forest’

• Line 545: Past tense needed: “edit” should be replaced by “edited”

Thank you, we replaced it.

• Line 547: The sentence is difficult to follow (too many “to”): “ …to calculate the monthly water deficit to obtain the annual MCWD, to use the monthly spatial average (1985-2020) evapotranspiration from TerraClimate”.

Thank you, we modified to "We modified the script to calculate the MCWD using the monthly spatial average evapotranspiration (1985–2020) from TerraClimate51, rather than a single average value for all pixels."

Conclusion:
The manuscript assesses how forest recovery and carbon accumulation are affected along a forest fragmentation gradient, providing insights into the resilience of the Atlantic forests and highlighting the importance of landscape consideration in forest policy and management. I recommend this paper for publication after minor revisions.

Reviewer #2 (Remarks to the Author):

In this paper the authors combine a number of remote sensing datasets (land classification from MapBiomas, biomass from ESA) to model the recovery of aboveground carbon stocks through time relative to old-growth forests in the Brazilian Atlantic Forest. They find regional differences in recovery rates, and most importantly that landscape connectivity is a very important driver of recovery, with secondary forests in highly connected landscapes recovering far more swiftly than those in low-connectivity landscapes. The paper reads well, tackles an important question at a regional scale with useful implications for restoration planning, and is presented really nicely, good job!
I like the paper, and the way the findings are represented, but I have a couple of important points that need to be addressed.

We thank the reviewer for the thorough and thoughtful evaluation of our manuscript. We are grateful for the positive assessment of the study's relevance, clarity, and contribution, as well as for the constructive comments provided. Below, we address the reviewer important points in detail.

- Medians when modelling recovery – I have looked through the methods of this paper, and the previous papers cited in this one that use the same methodology, and I cannot find any justification for using only the median value for each potential SF age when fitting the growth curve. You state that you have ~400,000 SF polygons, so why not put all the data into the growth model, instead of reducing all that data/variation down to just 1 summary value per potential age year? This confuses me. There are arguments you could make (e.g. remote sensing biomass estimates are inaccurate so this balances it out), but I don't see any justification in the paper for only using medians. I think a clear and convincing argument needs to be presented for why all the analysis relies on median values, and the raw data needs to be shown somewhere (at least in the supplementary).

We thank the reviewer for highlighting the important issue of using median above-ground carbon (AGC) values when fitting recovery curves. We agree that the rationale needed to be stated more clearly, and we have now added a justification in the revised manuscript.
Our decision to use the median AGC for each stand-age class was motivated by following considerations: Remotely sensed AGC estimates are known to suffer from heteroscedastic errors, age-dependent saturation, and systematic biases, particularly in high-biomass

tropical forests (e.g., saturation effects described in Araza et al. (2022). These issues can inflate variance and distort the fitted recovery trajectories when raw pixel- or polygon-level data are used directly. Using medians reduces the influence of extreme or biased values in early or late successional stages, producing more stable and representative age-class estimates. Because each age-based region group contains thousands of polygons, their empirical distributions are highly skewed and contain occasional artefacts related to misclassification, fragment shape and misalignment or local disturbance events. For such large, skewed samples, the median is a well-established estimator of the central trend that is robust to outliers and model noise. This is particularly important when the goal is to characterize the general recovery pattern rather than model small-scale spatial variability.
To address the reviewer's request for transparency, we have
added boxplots (Supplementary Figure 7, pasted below) showing the full distribution of AGC values for each age and region in the Supplementary Information.
We have also added a sentence in the Methods clarifying the rationale for using the median:
"We used the median aboveground carbon from thousands of fragments to reduce the potential biases due to fragment shape, pixel misalignment and saturation effect from the biomass data[42]. (Lines 702-704)

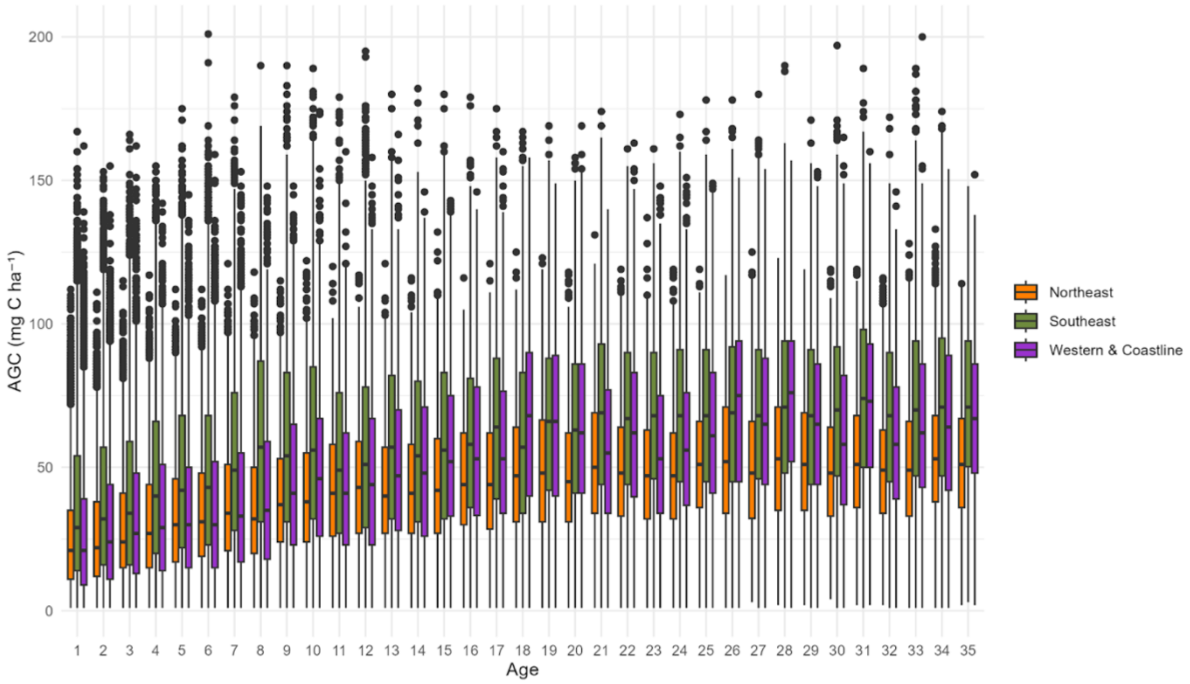

Supplementary Figure 7. Boxplot with the Aboveground Carbon (AGC) distribution by secondary forest age groups and Atlantic Forest regions.

- 3 regions from K means-clustering – I'll start by saying I am not an expert on the Brazilian Atlantic Forest, so I am happy to be proved wrong here. However, I did not read any justification for the K-means clustering approach to create different regions. Maybe you already tested a number of different potential K's (e.g. 2, 3, 4, 5) and found that k= 3 did the best job, if so great, but if not, I do not understand why there are 3 pre-determined regions. Perhaps these regional groupings are common/well known, if so you should include information about the regions and why they have been used. If not, you need to include more information on how you did the k-means clustering and how you selected k = 3. I did a quick search of recent papers on the Brazilian Atlantic Forest and found none referring to 3

regions similar to those presented in this paper. I'm not discrediting the three regions, just find that currently there is not an adequate explanation as to how they were chosen, so if the regions are to be kept a proper explanation will need to be added. I also think a value and growth curve for the entire Atlantic Forest would be a useful addition too.
Some additional specific comments:

We thank the reviewer for raising this important point. We agree that the rationale for the regionalisation approach needed to be explained more clearly, and we have now expanded this section in the revised manuscript as requested by reviewer 1 as well.
Our motivation for using k-means clustering was to identify environmentally similar subregions within the Brazilian Atlantic Forest that share similar climatic and topographic conditions, factors known to strongly influence forest structure, species composition, and successional trajectories. The Atlantic Forest spans a broad latitudinal and climatic gradient from Tropical to Subtropical regions, encompassing climatic gradients ranging from wetter and cooler in the south and in high elevations, hotter and relatively flatter terrain in the western and in lowland coastal areas, and hotter and drier in the northeast. Supplementary Table 1 shows the characteristics of each region determined by the kmeans clustering described in the Methods.
Although k-means regionalisation of the Atlantic Forest is not standardized, prior work has identified three major functional regions and climatic diversity regions that broadly correspond to our clustering results[1,2], reinforcing that the domain can be meaningfully grouped into three large-scale functional/bioclimatic units. We also added a figure showing the climatic and topographic variables maps used in the kmeans clustering definition in the supplementary material along the maps of clustering with k=3, k=4 and k=5. By visually analysing the clustering and the environmental variables used in its definition we believe the cluster k=3 reflects the broader regionalization of the Atlantic Forest and broadly align with the three major functional regions found in previous studies.
We edited the methods section to include the justification: "To determine the input variables for clustering the pixels based on the relationship with the AGC, we first calculated a correlogram to analyse collinear relationships between the environmental variables using a rank-based Spearman correlogram (Supplementary Figure 8). Variables with correlations exceeding ±0.5 were excluded to minimize multicollinearity. The final set of predictors for the k-means clustering included mean maximum temperature, mean maximum cumulative water deficit (MCWD), and terrain slope. The number of clusters was set to three to capture the dominant bioclimatic gradients of the Atlantic Forest. This choice reflects the broad environmental heterogeneity of the biome, which spans cooler and wetter southern and high elevation areas, hotter and flatter western and coastal lowlands, and hotter and drier conditions in the northeast. Clustering solutions with higher number of clusters (k=4 and k=5) primarily subdivided these broad regions without revealing clearly distinct climatic domains (Supplementary Figure 9). The resulting three regions were consistent with major functional and bioclimatic divisions of the Atlantic Forest reported in previous studies[56,57]." (Lines 641-658)

1. Silva, J. L. A., Souza, A. F. & Vitória, A. P. Mapping functional tree regions of the Atlantic Forest: how much is left and opportunities for conservation. *Environmental Conservation* **49**, 164–171 (2022).

2. Brown, J. L. *et al.* Seeing the forest through many trees: Multi-taxon patterns of phylogenetic diversity in the Atlantic Forest hotspot. *Diversity and Distributions* **26**, 1160–1176 (2020).

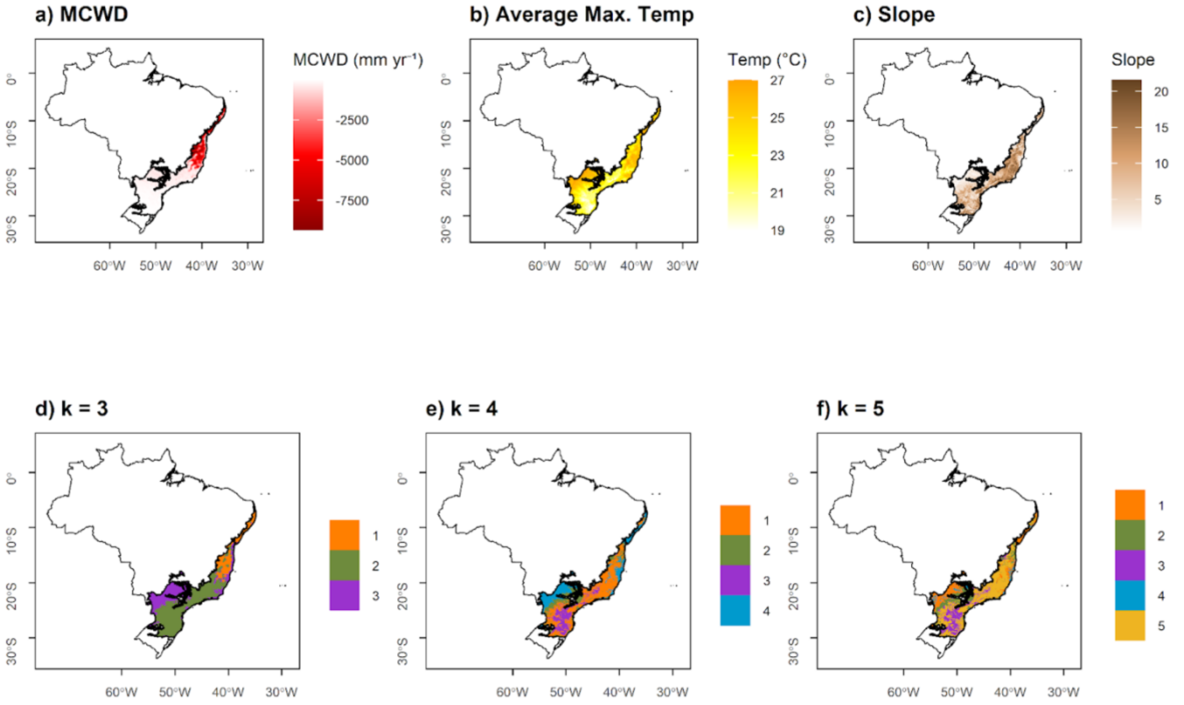

Supplementary Figure 9. Maps showing the spatial variation of the (a) average maximum cumulative water deficit (MCWD) between 1985-2020; (b) the average maximum temperature between 1985-2020; (c) terrain slope; and the k-means clustering test with (d) 3 regions, (e) 4 regions and (f) 5 regions.

Following the reviewer's suggestion, we have now added a recovery curve for the entire Atlantic Forest in Figure 1 along the regional curves. This provides a useful baseline comparison while our regional curves retain the advantage of capturing environmentally driven differences in growth rates.

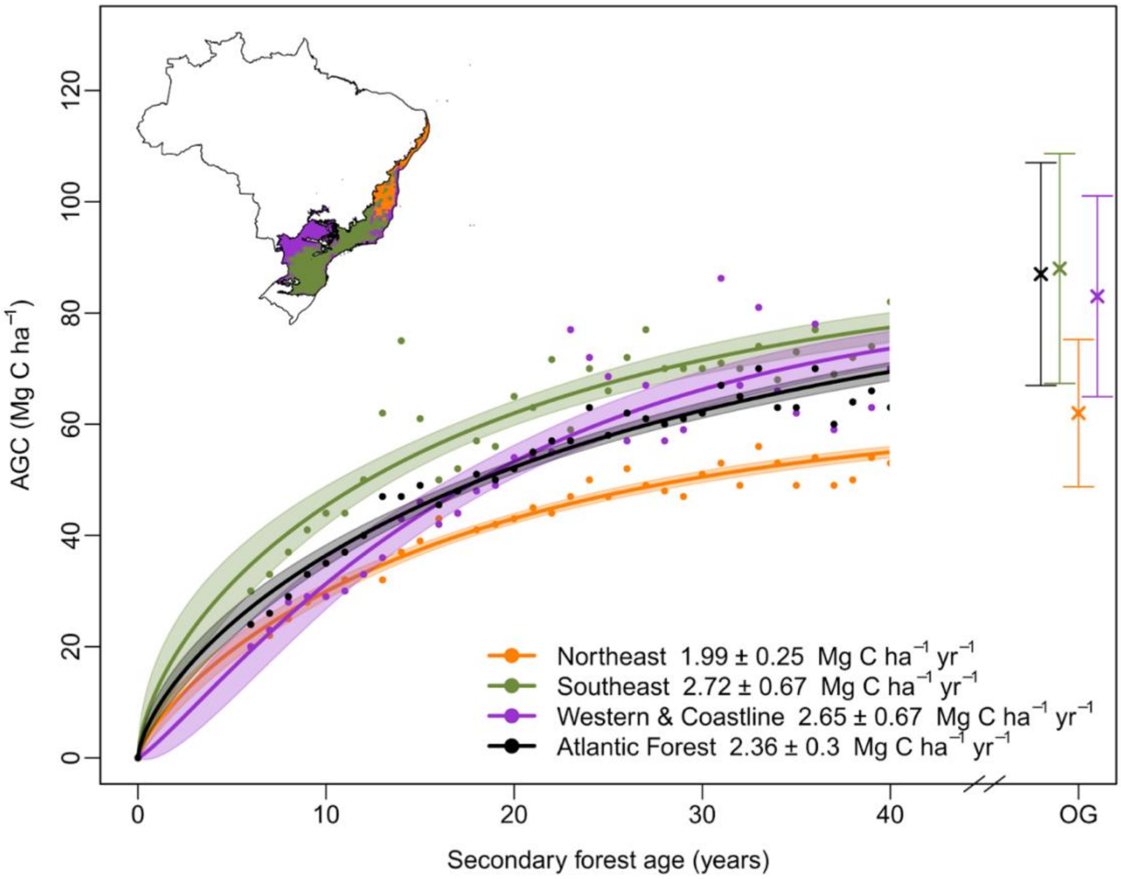

Fig. 1 | Atlantic secondary forest aboveground carbon accumulation with increasing age for each environmental region and for the whole Atlantic Forest. The environmental regions are grouped according to similarities in average annual maximum cumulative water deficit (MCWD; mm yr^{-1}) between 1985-2020, average maximum temperature between 1985-2020 from the ERA5 dataset[20], and terrain slope from the SRTM dataset[21]. Shading denotes the 95% confidence interval from the Chapman-Richards regrowth model[22], based on the median value of the initial aboveground carbon for each age (dots in the figure). Crosses denote the median aboveground carbon of old-growth (OG) forests for each region (may include forests older than 35 years due to the data availability), and the uncertainty line represents the median and 95% CI. The legend shows the 20-year average growth rate of secondary forests in each region and the average confidence interval (±95% CI).

• L27 – can you give this as a % increase or something relative to make it more tangible to the reader?

Yes, we added the % increase, now the sentence is "We modelled carbon stocks and found that full protection of secondary forests as of 2020 could increase stocks by 35% (132 Tg C) by 2030." (Lines 27-29)

• L64 - *forest
Corrected.

• 92-97 – pretty lengthy opening sentence, suggest splitting into two

Thank you. We split the sentence into two as suggested "To model the regrowth rates of secondary forests across the Brazilian Atlantic Forest (BAF) we used samples from the MapBiomas annual land cover product (collection 7.1)[18] to identify the secondary forest fragments and their respective ages from 1986 to 2020. These data were then associated with the biomass values extracted from the European Space Agency Climate Change Initiative (ESA-CCI) Aboveground Biomass product for 2020 (version 4)[19]." (Lines 95-100)

• 113-117 – this is phrased slightly confusingly, 'to leave the secondary forests standing in 2020' – do you mean that it will take 47 years from 2020 for secondary forests to reach 95% of old-growth AGC, or just that it takes 47 years in total for a secondary forest to regrow to 95% of old-growth AGC – if the later, I suggest phrasing it more clearly

Thank you, we changed the phrase to "Based on the estimated growth curves, secondary forests would require approximately 47, 62, and 52 years to reach the median aboveground carbon (AGC) of old-growth forests in the Western and Coastline, Southeast and Northeast regions, respectively, assuming that prevailing environmental conditions remain unchanged."(Lines 117-122)

• 159 – why group them into categories – why not just model the continuous effect of connectivity on regrowth?

We categorized forest connectivity into discrete classes for the construction and interpretation of regrowth curves using the Chapman-Richards (CR) modelling approach under contrasting landscape contexts. The categorical framework allows a direct comparison of regrowth trajectories among clearly defined connectivity scenarios (very low, moderate, and high) and provides boundary conditions required by the CR formula. This approach facilitates the generation of regrowth rates that can be applied beyond the research context, including in policy and restoration planning.
It is important to note that the continuous effect of connectivity on aboveground carbon (AGC) stocks of secondary forests was assessed separately using a multilinear analysis with a Generalized Least Squares (GLS) approach. The categorical approach was specifically chosen for the regrowth curves to meet the modelling requirements and practical application needs.

• 216 – useful to put a % increase to contextualise it – e.g. increase 35% to 517

Thank you, we added the percentage increase and now the sentences are: "If these forests are preserved, the projected carbon stock would increase by approximately 35% to 517 (CI: 496, 539) Tg C by 2030 and 66% to 636 (CI: 617, 656) Tg C by 2050. In Scenario 2, the initial carbon stock in 2020 was estimated at 321 (CI: 307, 336) Tg C from secondary forests older than eight years. If these forests alone are preserved, the projected carbon stock would

increase by approximately 18% to 379 (CI: 366, 393) Tg C by 2030 and by approximately 36% to 435 (CI: 423, 447) Tg C by 2050." (Lines 225-231)

• 238 – this is the first we are hearing of Scenario 3 – I am assuming it was a previous version that was removed

Thank you, we removed the reference to Scenario 3.

• Table 1 – if you are sticking with the 2 scenarios, this table is not necessary, since you report all the values within the text. I suggest removing it or moving to supplementary

Thank you, we moved the table to supplementary.

• 364 – To me this does not flow right by introducing new results and figures in the middle of your discussion. I think these sections need to be moved up in the main paper

In this section we discuss the implications of secondary forest in climate mitigation goals. We moved the sentence about the 2020 estimates of carbon stocks and Figure 5 to the end of result section: "The carbon potential of Brazilian Atlantic secondary forests".
"We further estimated that, in 2020, gross carbon emissions from deforestation in old-growth forests in the Brazilian Atlantic Forest region were approximately 10.3 Tg C yr^{-1} (see Methods for details). Meanwhile, the net aboveground carbon sink of secondary forests between 2019 and 2020 reached 13.3 Tg C yr^{-1} (Fig. 5), which would offset these gross emissions from old-growth forest loss. This additional analysis, independent of the scenarios described above, was based on the standing secondary forests in 2019 and 2020 and provides a snapshot of short-term carbon stock changes in the biome." (Lines 241-249)

• 366-368 – and I think I am right in saying that all your presented results are for AGB only? So SFs would have even less carbon compared to old-growth if accounting for below-ground carbo also

Yes, we agreed. We added "above and belowground biomass and soils". (Line 398)

• 407 – again, it's a nice figure, but not one that should be introduced in the discussion. I suggest this one gets moved to supplementary
Thank you for the suggestion, we moved this figure to the supplementary.

• 437 – you could also allude to new datasets that will be soon be released that should help with this (e.g. BIOMASS)

Thank you, we added the new missions such as BIOMASS and NISAR in the text : "Future studies should explore the integration of higher-resolution remote sensing datasets and active remote sensing techniques, such as LiDAR and new missions (e.g. BIOMASS, NISAR). For instance, BIOMASS is a P-Band sensor and therefore might help to reduce potential saturation effect in high-biomass tropical regions[45], however old-growth baselines are needed to help build these recovery curves. Other missions such as NASA-ISRO Synthetic Aperture Radar (NISAR) might also prove helpful to further improving aboveground biomass

estimates as they have higher resolution. Therefore, refining land cover classification algorithms, improving biomass estimates derived from earth observation data, and incorporating field validation efforts will be essential for enabling that such data to be used effectively by end-users such by restoration monitoring communities[47]. " (Lines 482-495)

• 551 – I'm not hugely familiar with the Brazilian Atlantic Forest, but interested in why you split the biome into three regions, what's the rationale for this? Is it generally accepted that there are 3 distinct regions? Did you test other numbers on the clustering?

Thank you. We added the justification in the manuscript methods section: "To determine the input variables for clustering the pixels based on the relationship with the AGC, we first calculated a correlogram to analyse collinear relationships between the environmental variables using a rank-based Spearman correlogram (Supplementary Figure 8). Variables with correlations exceeding ±0.5 were excluded to minimize multicollinearity. The final set of predictors for the k-means clustering included mean maximum temperature, mean maximum cumulative water deficit (MCWD), and terrain slope. The number of clusters was set to three to capture the dominant bioclimatic gradients of the Atlantic Forest. This choice reflects the broad environmental heterogeneity of the biome, which spans cooler and wetter southern and high elevation areas, hotter and flatter western and coastal lowlands, and hotter and drier conditions in the northeast. Clustering solutions with higher number of clusters (k=4 and k=5) primarily subdivided these broad regions without revealing clearly distinct climatic domains (Supplementary Figure 9). The resulting three regions were consistent with major functional and bioclimatic divisions of the Atlantic Forest reported in previous studies[56,57]. The k-means clustering classification was performed on the Google Earth Engine platform." (Lines 641-658)

• 572- how come you grouped the old growth and secondary forests into one mask for the fragmentation analysis, wont this put secondary and old-growth forest on the same level in terms of connectivity value? It's likely that the proportion of surrounding forest that is old growth vs secondary will have a strong impact on biomass recovery (e.g. an area that is surrounded by 70% forest, all of which is old-growth, is likely to be getting more dispersal etc. than an area surrounded by 80% forest but most of this is secondary?)

We grouped old-growth and secondary forests into a single mask for the fragmentation/connectivity analysis to capture the overall influence of landscape connectivity on secondary forest regrowth. If we had used only a secondary forest mask, the metric would not account for connections to old-growth forests, which are critical sources of propagules and ecological influence in naturally regrowing forests. The FAD metric, as applied, reflects both the landscape connectivity/fragmentation and the local forest area density, thereby integrating the effects of proximity to any forest type, whether old-growth or secondary. This approach allows us to assess the overall structural context of the landscape that governs regrowth dynamics.

• 577 – what is the dominant value? Median? Better to state this explicitly

Thank you for your comment. Yes, it is the median. We now replaced the word dominant to 'median'.

- 588 – equation format has not come through correctly in the PDF

Thank you, we checked and it is working when we generate the PDF.

Response to the Reviewers
The responses from the author are highlighted in blue colour.

Reviewer #1 (Remarks to the Author):

I thank the authors for the careful clarification.

Regarding clustering, I was curious whether the authors used specific techniques to identify natural grouping in the data (e.g. Elbow method, silhouette method etc.). These would provide more robust justification to determine an optimal number of clusters, as they aim to maximise cohesion within groups and separation among them. But I think the rationale for taking 3 clusters has been well explained, as I understand the intention is to highlight known bioclimatic regions.

Overall, I am satisfied with the Authors' response and revisions. All the concerns I raised were thoughtfully addressed and the clarity of the manuscript has been substantially improved. I recommend this paper for publication.

We thank Reviewer #1 for the positive assessment and for the additional methodological note regarding clustering techniques (e.g., Elbow method, silhouette method). We agree that such approaches provide a more formal statistical justification for the choice of cluster number. As the reviewer acknowledges, our rationale for selecting three clusters was guided by the intention to reflect known bioclimatic regions rather than to identify purely data-driven groupings, and we are glad this reasoning was clear. We appreciate the reviewer's understanding of this decision and their recommendation for publication.

Reviewer #2 (Remarks to the Author):

The authors have done a good job responding to all my comments, making some necessary changes and improvements, and explaining their analytical decisions. I am happy that the manuscript is now ready for publication.
Below are just a small number of changes I noticed were required when re-reading the manuscript in full:
L373 – stopping not 'stop' - done
L383-385 – you should also add in that it takes far longer to achieve old-growth biodiversity (if at all) than it does carbon stocks - done
L407 – y? typo here I think - done
Fig 1 – thank you for including the biome-wide growth curve as I suggested. Looking at the figure, I understand if the authors would rather remove this black curve from the main text figure to keep continuity with the three models presented in other figures. If so, please put the biome-wide curve in the supplementary, and revert to just the three regions in Fig 1 (I do not mind which – the authors can choose what option they prefer)

We sincerely thank both reviewers for their time and constructive engagement throughout the review process. We are pleased that both reviewers are now satisfied with the manuscript and recommend it for publication.

We thank Reviewer #2 for carefully re-reading the revised manuscript and for their positive recommendation. We are pleased to confirm that the minor textual corrections identified have been addressed:

- L373: Corrected to 'stopping'.
- L383–385: Added text noting that it takes far longer to achieve old-growth biodiversity (if at all) than it does carbon stocks.
- L407: Typo corrected.
- Fig. 1: We have chosen to retain the biome-wide growth curve in Fig. 1 alongside the three regional models. We believe this provides useful context directly in the main text and avoids the need to renumber supplementary figures at this stage. We hope the reviewer finds this an acceptable choice given their indication that either option was suitable.
